# Cervicovaginal Microbiota and Biogenic Amine Metabolic Shifts in HPV-Associated Cervical Disease

**DOI:** 10.3390/cancers18121931

**Published:** 2026-06-13

**Authors:** Natalie M. Meléndez-Vázquez, Nataliya Chorna, Cecilia Noecker, Andrea P. Cortes-Nazario, Josefina Romaguera, Filipa Godoy-Vitorino

**Affiliations:** 1 Department of Microbiology and Immunology, School of Medicine, University of Puerto Rico-Medical Sciences Campus, San Juan, PR 00936, USA; natalie.melendez2@upr.edu (N.M.M.-V.); andrea.cortes1@upr.edu (A.P.C.-N.); 2 Department of Biochemistry, School of Medicine, University of Puerto Rico-Medical Sciences Campus, San Juan, PR 00936, USA; nataliya.chorna@upr.edu; 3 Department of Biological Sciences, Minnesota State University—Mankato, Mankato, MN 56001, USA; cecilia.noecker@mnsu.edu; 4 Department of Obstetrics and Gynecology, School of Medicine, University of Puerto Rico-Medical Sciences Campus, San Juan, PR 00936, USA; josefina.romaguera@upr.edu

**Keywords:** cervicovaginal microbiome, biogenic amines, tyramine, cervical cancer

## Abstract

**Background**: Cervical cancer is primarily caused by the human papillomavirus (HPV), with persistent infections progressing to low- (LGSIL) and high-grade (HGSIL) lesions. Emerging evidence indicates that the cervicovaginal microbiota influences HPV persistence and disease progression, although the underlying metabolic mechanisms remain unclear. Therefore, we assessed the relationship between the cervicovaginal microbiota and the metabolic milieu in women with cervical dysplasia and HPV infections. **Methods**: We recruited 36 non-menopausal, non-pregnant women who were classified as negative, LGSIL, or HGSIL based on pathology and HPV results. Cervical swabs were collected for genomic DNA extraction to characterize bacterial communities using 16S rRNA sequencing and to perform HPV genotyping. Cervical lavages were collected for untargeted metabolomic profiling using Gas Chromatography-Mass Spectrometry. Integrative multiomic analysis was performed using the MIMOSA2 pipeline. **Results**: Although bacterial community structure was not different between groups, women with HGSIL had higher richness and exhibited a higher abundance of *Prevotella bivia*, *Prevotella buccalis*, and *Lachnospiraceae G-9 oral taxon 924*. Lesion-positive samples had shifts in tyramine and putrescine, biogenic amines linked to cancer development. Specifically, *Pseudomonas* was identified as a potential contributor to tyramine oxidation. **Conclusions**: Cervical lesions and HPV risk are associated with shifts in the cervicovaginal microbial metabolic milieu, highlighting the role of low-abundant anaerobic bacteria. Despite the small sample size, biogenic amines were associated with anaerobic taxa and microbial dysbiosis. These findings warrant further assessment of microbial-derived metabolites and their potential to promote tumor progression by driving a pro-inflammatory, metabolically altered microenvironment.

## Simple Summary

Biological factors, such as the cervicovaginal microbiome, may influence HPV infection persistence and disease progression, leading to cervical cancer development. Here, we showed the potential of putative bacteria to drive shifts in biogenic amine levels, which favor an inflammatory environment, ideal for HPV persistence. Our study provides exploratory insights into possible microbial–molecular mechanisms that may be important to cervical disease.

## 1. Introduction

With an estimated 660,000 new cases and 350,000 deaths worldwide in 2022, cervical cancer remains the 4th most common cancer in incidence and mortality in women [1]. Compared to non-Hispanic White women, Hispanics have a 36% higher incidence rate of cervical cancer [2]. Incidence among women living in Puerto Rico has increased from 9.0 to 13.0 per 100,000 person-years [3], likely due to socioeconomic factors that limit access to healthcare, reduced follow-up for abnormal results, lower screening rates, and likely new human papillomavirus (HPV) not covered by the vaccine [4,5,6].

A key factor in cervical disease progression is the persistence of high-risk HPV infections. As the etiological agent of cervical cancer, HPV is a key element in the complex process of carcinogenesis [7]. Although over 200 HPV serotypes have been identified, most infections are asymptomatic and self-limiting. Clinical concern focuses on a subset of mucosal high-risk HPVs capable of persistent infection and cervical colonization. Cervical lesions are characterized by cytology using the Bethesda System, which identifies Pap smear results as: (1) negative for intraepithelial lesion or malignancy (NILM), (2) atypical squamous cells of undetermined significance (ASCUS), (3) low-grade squamous intraepithelial lesion (LGSIL), and (4) high-grade squamous intraepithelial lesion (HGSIL) [8]. Additionally, the American Society for Colposcopy and Cervical Pathology (ASCCP) developed a consensus screening guideline for cervical abnormalities based on biopsy results as cervical intraepithelial neoplasia (CIN) 1, CIN2, and CIN3 [9].

Recent evidence suggests the cervicovaginal microbiota influences viral persistence and cancer progression [10,11,12,13]. The vaginal canal has an epithelial barrier that, under the influence of estrogen, promotes *Lactobacillus* dominance [14]. These communities are classified into distinct community state types (CSTs) depending on the dominant bacterial species: CST I (dominated by *Lactobacillus crispatus*), CST II (*Lactobacillus gasseri*), CST III (*Lactobacillus iners*), CST IV (diverse group; low *Lactobacillus*), and CST V (*Lactobacillus jensenii*) [15]. The vaginal microbiota in White women is typically dominated by CST I, whereas Black and Hispanic women more frequently exhibit CST III and CST IV, with a higher prevalence of anaerobic taxa [15]. In Puerto Rican women of reproductive age, the cervical microbiota is predominantly CST III [16]. Pregnancy does not alter CST III dominance, although an increased prevalence of CST I has been observed among pregnant women in Puerto Rico [5]. On the other hand, CST IV is more common in women with menopause, along with increased risk of bacterial vaginosis and recurrent urinary tract infections [5]. Studies have shown elevated pro-inflammatory cytokines, such as IL-1β, IL-15, and TNF-α, in dysbiotic or non-*Lactobacillus*-dominated vaginal communities [17,18].

The role of microbial-derived metabolites in cancer biology is increasingly recognized as a critical factor in tumor initiation and progression. Bacterial metabolites, including biogenic amines, can modulate the local microenvironment by promoting inflammation, altering epithelial integrity, and influencing host immune responses, thereby contributing to malignant tumor development [19]. Multi-omics techniques, such as metagenomics and metabolomics, are proving to be promising for unraveling host–microbe interactions. Shifts within the cervicovaginal microbiota due to dysbiosis can alter the microbial metabolome. For instance, a shift from a *Lactobacillus*-dominated environment to anaerobic taxa, as seen during bacterial vaginosis, is associated with an increase of polyamines such as putrescine, cadaverine, and tyramine [6,20,21]. Recently, spermidine, a byproduct of putrescine degradation, was identified as one of the most differential metabolites in a cohort of 156 patients with and without cervical lesions [22]. Additional comparisons between LGSIL and HGSIL patients revealed a negative correlation between *Gardnerella* and succinic acid [22]. A previous study has also assessed microbial and metabolome changes between HPV positive and HPV negative patients, where HPV positive women had higher concentrations of biogenic amines [23]. Specifically, HPV positive non-pregnant women predominantly characterized by CST III showed the highest abundance of these metabolites [23]. Among Puerto Rican women, who exhibit a high prevalence of cervicovaginal dysbiosis, the metabolic mechanisms through which microbial communities may influence cervical disease and high-risk HPV persistence remain poorly understood.

Therefore, this study aimed to assess the associations between the cervicovaginal microbiota and the metabolic milieu in women with cervical disease and high-risk HPV infections, as well as identify possible microbial metabolic mechanisms underlying those associations.

## 2. Materials and Methods

### 2.1. Cross-Sectional Study Design and Sample Collection

Recruitment for this cross-sectional study was conducted in compliance with the Human Subjects Protection Office and approved by the Institutional Review Board (IRB) of the University of Puerto Rico (UPR)-Medical Sciences Campus (Protocol #1050114, Streamlyne #2290033153), with biosafety approval under protocol #94620. The IRB protocol was originally approved on 24 June 2014 and has been consistently renewed, remaining active to date. Staff involved in recruitment, sample collection, and processing were certified with the CITI Program in Responsible Conduct of Research, Social and Behavioral Research Best Practices for Clinical Research, HIPAA, and Protection of Human Subjects.

Women aged 21 to 60 years who did not meet the exclusion criteria were recruited between November 2017 and May 2019 at the UPR-Medical Sciences Campus gynecology and obstetrics clinics at San Juan, Puerto Rico. The exclusion criteria, selected based on the Manual of Procedures of the Human Microbiota Project protocol [24], included the following: (1) hysterectomy, (2) antibiotic use within the last 1–3 months, (3) candidiasis, (4) history of regular urinary incontinence, (5) active urinary tract infections, (6) cervicovaginal irritation at the time of screening, (7) treatment for or suspicion of prior toxic shock syndrome, (8) active sexually transmitted diseases (STIs) other than HPV. All subjects received and signed informed consent and HIPAA forms that detailed the risks and benefits of the study and discussed the sampling procedures. Patients also completed a questionnaire with basic demographics, medical history, lifestyle factors, and sexual behaviors. In addition, cervical cytology and pathology reports from their medical records were obtained to classify patients by cervical disease status. For this specific analysis, diseases status was categorized according to the Bethesda System as: (1) Negative for intraepithelial lesion or malignancy without HPV (NILM HPV−), (2) NILM with an HPV positive result (NILM HPV+), (3) low-grade squamous intraepithelial lesion with HPV positive status (LGSIL), and (4) high-grade squamous intraepithelial lesion with HPV (HGSIL) [25]. No colposcopy nor biopsy was performed if a patient had NILM with low-risk HPV. Biopsy results replaced cytology diagnosis only for NILM high-risk HPV, LGSIL, and HGSIL patients.

Posterior fornix swabs and cervical lavages (CVL) were collected from 46 patients, and after careful assessment, 7 samples were excluded due to menopause status and 3 samples due to lack of metabolomics data. Hence, the final subset of samples analyzed further downstream comprised 36 non-menopausal, non-pregnant, and HIV-negative patients grouped as follows: NILM HPV− (n = 8), NILM HPV+ (n = 12), LGSIL (n = 5), and HGSIL (n = 11). For sampling, a speculum was inserted for access to the cervix during gynecological examination, where swab samples were collected using the Puritan Sterile Standard Cotton Swab with Dry Transport System (Puritan Medical Products, Guilford, ME, USA) and later placed in bead tubes with 750 µL of MoBio buffer (MoBio PowerSoil kit, MoBio, Carlsbad, CA, USA) [24]. Pure certified nuclease-free water (Growcells, Irvine, CA, USA) was inserted into the vaginal canal, where approximately 10–15 mL of CVL was collected and stored in clean 50 mL Falcon collection tubes. All samples were temporarily stored for 4 h in a cooler with ice packs before transportation to the lab. Upon arrival, aliquots were prepared following sterile techniques under a biological hood and were stored at −80 °C until further experiments.

### 2.2. Genomic DNA Extraction and 16S rRNA Sequencing

Genomic DNA (gDNA) extraction was performed on posterior fornix swabs with the QIAGEN DNeasy PowerSoil Kit (QIAGEN LLC, Germantown Road, MD, USA) following the manufacturers protocol with additional modifications: (1) a combination of solutions C2 and C3 (100 µL each) was added to the sample and later incubated at −20 °C for 10 min, (2) 1200 µL of C4 were added to the supernatant, (3) lysate was placed in a spin filter membrane using a QIAvac Vacuum System (QIAGEN LLC, Germantown Road, MD, USA), and (4) elution was made with 100 μL of warmed (55–65 °C) C6 solution or pure certified nuclease-free water. To increase gDNA yield, C6 solution or pure certified nuclease-free water remained on the spin filter for 5 min before final vacuum. DNA quantifications were measured with the Qubit 1X dsDNA HS (High Sensitivity) Assay Kit (Thermo Fisher, Waltham, MA, USA) and the Qubit 2.0 Fluorometer (Thermo Fisher, Waltham, MA, USA).

For bacterial identification, the V4 hypervariable region of the 16S ribosomal RNA marker gene was amplified using the universal bacterial primers 515F (5′-GTGCCAGCMGCCGCGGTAA-3′) and 806R (5′-GGACTACHVGGGTWTCTAAT-3′) as described in the Earth Microbiome Project (https://earthmicrobiome.org/protocols-and-standards/16s/, accessed on 8 November 2019). Amplicons were outsourced to Argonne National Laboratory (Lemont, IL, USA) to be sequenced with Illumina Miseq using a 2 × 250 base paired-end approach. Raw reads were uploaded in the QIITA study ID 12871, sandbox ID 7969 (https://qiita.ucsd.edu/study/description/12871, accessed on 10 January 2025), and are publicly available at the European Nucleotide Archive Project (ENA) under the accession number PRJEB51893, ERP136546.

### 2.3. HPV Genotyping

Extracted DNA was also used for HPV serotype detection and identification with the SPF10-LiPA assay (Labo Biomedical Products, Rijswijk, The Netherlands, licensed Innogenetics Technology), as previously described [5]. Identified mucosal HPVs are classified as low-risk (6, 11, 34, 40, 42, 43, 44, 53, 54, 70, 74) or high-risk (16, 18, 31, 33, 35, 39, 45, 51, 52, 56, 58, 59, 66 and 68/73) serotypes. In this paper, we categorize our results as either HPV negative or HPV positive (has either a coinfection of low- and high-risk serotypes or only high-risk HPVs).

### 2.4. Analyses of Microbiota in Community State Types

The cervicovaginal microbiome is classified into five main community state types (CSTs): CST I—dominated by *Lactobacillus crispatus*, CST II—dominated by *Lactobacillus gasseri*, CST III—dominated by *Lactobacillus iners*, CST IV—dominated by anaerobic bacteria (non-*Lactobacillus*), and CST V—dominated by *Lactobacillus jensenii*. The Vaginal Community State Type Nearest Centroid Classifier (VALENCIA) is a nearest-centroid-based algorithm that classifies the vaginal species into the five CSTs described previously and additional sub-CSTs [26]. Hence, each individual sample is assigned a CST based on its similarity to a set of thirteen reference centroids. This algorithm was run locally in the terminal using python3 and the VALENCIA data package downloaded from GitHub (https://github.com/ravel-lab/VALENCIA, accessed on 23 January 2025) using the formatted species feature table. The CST prevalence plot was generated using the *ggplot2* R package (version 3.5.2), while the 95% confidence intervals for these values were calculated using the BinomCI function from the *DescTools* package (version 0.99.60) [27]. To test for statistical significance, Fisher’s exact pairwise test was used.

### 2.5. Microbial Community Assessment

#### 2.5.1. Quality Control

The demultiplexed bacterial sequences were deposited on Qiita [28] for pre-processing with a Phred score offset of 30 and default parameters for split libraries (QIIMEq2 1.9.1). Sequences were trimmed at 250 base-pairs (bp) followed by a deblur workflow (deblur 2021.09) [29]. Amplicon sequence variant (ASV) species table was downloaded for downstream microbial analyses using a locally installed QIIME2 version (qiime2-amplicon-2023.9) [30]. Singletons, mitochondria, chloroplasts, and taxonomically unassigned sequences were removed before continuing further downstream. As described previously [5], taxonomy classification was performed using a Naïve Bayes approach with a minimum similarity threshold of 97% against a custom GreenGenes reference database that combines sequences from GreenGenes (Greengenes_13.8) [31], the Human Oral Microbiome Database [32], and cervicovaginal reference sequences from the Human Microbiome Database [33]. All samples underwent rarefaction at 5424 reads for an unbiased pipeline.

#### 2.5.2. Alpha and Beta Diversity Metrics

For community-level composition and structure, the Bray–Curtis Index was computed to assess dissimilarity between samples. Plot visualization was through a non-metric multidimensional scaling (NMDS) generated with the *phyloseq* (version 1.46.0) [34] and *ggplot2* [35] R packages. A complementary pairwise statistical test, Analysis of Similarities (ANOSIM), was used to assess the significance of group distances using the QIIME2 pipeline. The alpha diversity metrics Chao1, used to estimate the number of species (richness), and Shannon, which evaluates richness and distribution of species, were calculated and plotted at the feature-level through the *vegan* (version 2.7.1) [36], *phyloseq*, and *ggplot2* R packages. The non-parametric Kruskal–Wallis (KW) test was used to assess statistical significance within groups.

#### 2.5.3. Taxonomic Profiles and Microbial Biomarkers

Relative abundance profiles at phylum and genus levels were generated using the *phyloseq* and *ggplot2* R packages. Genus-level taxa were only shown for the top 25 most abundant bacterial communities. To assess and identify potential bacterial biomarkers, the machine-learning algorithm Random Forest was employed using MicrobiomeAnalyst [37]. The parameters selected for the phylum- and genus-level figures were 500 trees, 7 predictors, and randomness settings on. Additionally, to validate the model’s performance, the Out-of-Bag (OOB) error was calculated. Overall, the OOB error interpretation is as follows: <10% = excellent discrimination; 10–25% = good, but with some overlap; and >25% = the model may not be as reliable.

### 2.6. Protein Extraction and Quantification

Protein concentration from CVL samples was measured to concentrate proteins larger than 3 kilodaltons (kDa). This process allowed to concentrate up to 500 μL of CVL into a smaller volume (10 to 60 μL) with a higher protein concentration. A volume of 500 μL of pure certified nuclease-free water was loaded to the Nanosep^®^ Centrifugal Device column (Pall Corporation, Port Washington, NY, USA) to remove trace amounts of glycerin and sodium azide. After loading, centrifugation was performed at 14,000× *g* for 5 min for a total of two washes. CVL samples were loaded into the Nanosep^®^ device and centrifuged at 14,000× *g* for 15 min. If needed, this previous step was repeated using the same column and without removing the remnants between each centrifugation, allowing higher protein concentration to be obtained. Concentrated samples were recovered and transferred to a microtube following storage at −20 °C for short-term storage or −80 °C for long-term storage. Protein concentration was measured with 1 µL of CVL sample using the Nanodrop at 280 nm.

### 2.7. Untargeted Metabolomics

#### 2.7.1. Metabolite Extraction

One milliliter of CVL from each sample was mixed with 1 mL of extraction solution consisting of chloroform, methanol, and water, 2:5:2 *v*/*v*), vortexed for 1 min, and centrifuged at 13,000 rpm for 10 min at 4 °C. For quality control, 100 µL aliquots from each sample were pooled to generate a composite mixture from which 1 mL was transferred to a glass vial. Two extraction blanks were prepared using the same pure certified nuclease-free water used for CVL collection. Following centrifugation, supernatants were collected, transferred to glass vials, evaporated using a SpeedVac Concentrator (Savant AS160, Thermo Fisher Scientific, Waltham, MA, USA), and stored in zipped bags at −20 °C to prevent moisture until further analysis.

#### 2.7.2. Metabolite Derivatization and Gas Chromatography-Mass Spectrometry (GC-MS)

Before initiating the derivatization process, samples were removed from storage and allowed to warm up to room temperature for at least 15 min. Dried samples were first derivatized by methoxyamination by adding 30 μL of a 20 mg/mL methoxyamine hydrochloride solution in pyridine (Sigma-Aldrich, St. Louis, MO, USA) and incubated at 37 °C for 2 h. Trimethylsilylation was subsequently performed by adding 20 μL of N-methyl-N-trimethylsilyl-trifluoroacetamide with trimethylchlorosilane (MSTFA + 1% TMCS; Sigma-Aldrich, St. Louis, MO, USA) and incubated at 65 °C for 1 h. Samples were centrifuged at 14,000 rpm for 10 min at room temperature. A volume of 20 µL of each sample was aliquoted with 1 mM of the internal standard 2-Fluorobiphenyl in pyridine to an analytical glass vial with insert and processed by the GCMS-QP2010 gas chromatograph with the AOC-20i auto-injector in split mode (split ratio = 15) (Shimadzu Scientific, Columbia, MD, USA) under analytical conditions previously described [38,39]. Briefly, the chromatography conditions included an injection volume of 1 μL where the analytes were fractionated on a Restek RTX-5MS column (5% diphenyl and 95% dimethyl polysiloxane; 0.25 mm inner diameter, 0.25 μm D.F., 30 m; Restek, Bellefonte, PA, USA). The inlet temperature was set at 280 °C, while the interface temperature was maintained at 150 °C. The initial oven temperature was set at 100 °C with no hold time, then increased to 290 °C at 8 °C per minute, and held at 290 °C for 16 min. Helium was used as carrier gas at a constant linear velocity of 39 cm per seconds. Lastly, the mass-selective detector was operated in the electron impact (EI) ionization mode at an electron energy of 70 electronvolt (eV) with a quadrupole scan range of 35–700 *m*/*z*. For quality control, several samples, system suitability, and extraction processing blanks were incorporated during the derivatization process. To minimize bias, samples and blanks were randomized and distributed among the injections.

#### 2.7.3. Metabolite Library Preparation

Mass spectral library preparation was performed by initial searches of major chromatographic peaks using the GCMS Lab Solutions Software (Shimadzu Scientific, Columbia, MD, USA) equipped with the NIST14/2014/EPA/NIH database, resulting in the identification of 49 metabolic features for further analysis. The generated library underwent data deconvolution using the Automated Mass Spectral Deconvolution and Identification System (AMDIS). Here, overlapping peaks in the spectra of each metabolite were resolved and compared against the reference database spectra. Metabolite validation and retention within the library were confirmed by alignment of reference peaks with the corresponding sample peaks. Average peak intensities were calculated for each metabolite and normalized to the intensity of 1 mM of 2-Fluorobiphenyl as an internal standard to determine relative concentrations of each metabolic feature. Samples were normalized to the protein concentration of each individual sample.

#### 2.7.4. Multivariate and Univariate Analysis of Metabolomic Profiles

The data matrix containing the concentrations of 49 metabolites was processed using the web platform MetaboAnalyst 6.0 [40]. Once uploaded, metabolomics data underwent log-transformation (base 10) and Pareto scaling to improve pattern recognition. The dataset was evaluated using the unsupervised technique Principal Component Analysis (PCA) biplot, which interprets differences between samples and shows arrows indicating potential metabolites responsible for the differences seen between groups. In this method, variables that are close together have a high correlation, where the ellipses of the PCA illustrate 95% confidence region of the corresponding samples. Statistical significance was determined by employing a pairwise Permutational Multivariate Analysis of Variance (PERMANOVA) test. Normalized metabolite group averages were also visualized using a heatmap generated with Euclidean distance and the Ward clustering algorithm. Overall statistical significance in the differential abundance analysis was performed using Analysis of Variance (ANOVA) with multiple hypothesis correction applied via the Benjamini–Hochberg procedure, while pairwise comparisons were conducted with Tukey’s Honestly Significant Difference (HSD) test in RStudio (version 4.3.2). The normalized concentrations were downloaded from MetaboAnalyst for further downstream analysis. Lastly, metabolites were classified according to their main class using the Metabolomics Workbench webpage, section RefMet: A Reference list of Metabolite names (https://www.metabolomicsworkbench.org/databases/refmet/index.php, accessed on 15 April 2025). Percentages were determined and illustrated with a pie chart using GraphPad PRISM 8.4.0 (GraphPad Software, Boston, MA, USA, www.graphpad.com).

### 2.8. Functional Inference of the Microbiota

PICRUSt2 (Phylogenetic Investigation of Communities by Reconstruction of Unobserved States; version 2.6.2) is a software for predicting functional abundances based only on marker gene sequences [41]. The input data consisted of the clean species table and the reference hit sequences downloaded from the QIITA study sandbox. The output file of interest used in downstream analysis was the stratified metagenome predictions. Additional file formatting changes were made for it to be compatible with the MIMOSA2 webpage, including (1) header changes (sample to Sample, taxon to Gene, genome_function_count to GeneCountPerGenome, taxon_abun to OTUAbundanceInSample, and taxon_function_abun to CountContributedByOTU), (2) OTU sequences were changed for the corresponding taxa name, (3) shortening the taxon names to begin at family-level, (4) unique identifier was added to each taxon name as follows “OTU_#”, and (5) removing the “ko:” prefix from the Gene column.

### 2.9. Integrative Microbiome–Metabolome Analysis

Model-based Integration of Metabolite Observations and Species Abundances 2 (MIMOSA2) is an R package and web application for integration of microbiome and metabolome data [42]. It relates variations in metabolite measurements to microbial composition using reference databases to determine these capabilities. It uses the microbiome data and chooses a reference database (Kyoto Encyclopedia of Genes and Genomes (KEGG), AGORA, or RefSeq/EMBL_GEMs) to construct taxon-specific community metabolic models. These models predict the presence/absence of metabolic pathways based on uploaded microbiome data. Then, the community-level metabolic potential (CMP) is calculated based on the model’s predictions, highlighting differences in metabolite levels across samples and characterizing the capacity of community members to synthesize or degrade metabolites. These predictions are compared with the actual metabolome data uploaded to identify microbiome-governed metabolites and taxa contributors to metabolite variation. The overall model fit, explained with the R^2^ value, indicates how much of the variation in metabolite levels between the groups being analyzed is explained by the microbiome. This R^2^ value is then deconvolved into the contributions from each taxon, identifying specific taxa and metabolic reactions that explain variation in each metabolite across samples.

#### 2.9.1. MIMOSA2 Web Application Analysis

Input files required to run the MIMOSA2 web server (http://elbo-spice.cs.tau.ac.il/shiny/MIMOSA2shiny/, accessed on 21 May 2025) were the PICRUSt2 metagenome predictions with the modifications described previously and the metabolites’ normalized concentrations downloaded from MetaboAnalyst 6.0. The metabolite file incorporated the KEGG compound ID instead of the common metabolite name. Both the PICRUSt2 and metabolite files were filtered according to the groups being analyzed: (1) NILM HPV− (n = 8) with HGSIL (n = 11), (2) only HGSIL patients with normal weight (n = 4) and overweight/obese (n = 6) Body Mass Index (BMI) classifications, (3) only normal-weight patients to compare NILM HPV− (n = 6) with HGSIL (n = 4), and (4) LGSIL (n = 5) and HGSIL (n = 11) patients. The parameters selected on the MIMOSA2 platform for all group analyses were: (1) Metagenome: Taxon-stratified KO abundances (HUMAnN2 or PICRUSt/PICRUSt2), (2) PICRUSt KO genomes and KEGG metabolic models [43], (3) KEGG Compound IDs and Log transform metabolite values, and (4) rank-based estimation regression. The contribution results table and other output files were downloaded once the program ran. Specifically, the contribution results table identified the microbial contributors and putative genes that may be involved in the synthesis or degradation of a metabolite as determined from KEGG ortholog genes. Taxa with annotated genes associated with metabolite-producing reactions are classified as potential synthesizers, while taxa with genes linked to metabolite-consuming reactions are classified as degraders. This is indicated in the output by the numbers and identities of synthesis genes (NumSynthGenes, SynthGenes) and degradation genes (NumDegGenes, DegGenes), with contributing taxa interpreted from the presence of these genes and their explained variance (VarShare) for each metabolite. Following the software recommendations, metabolites consistent with microbial metabolic potential were characterized by their CMP scores, specifically highlighted by a positive model slope, a Variance Share (VarShare) value bigger than ±0.01, and a *p*-value < 0.1 (this looser *p*-value cutoff is used because the model is conservative and prone to false negatives).

#### 2.9.2. Combined Heatmap and CMP Graphical Visualization

Well-predicted metabolites, alongside normalized concentrations of these metabolites, and log-transformed relative abundance of contributor taxa were visualized in a heatmap using the R packages *tidyverse* version (2.0.0) [44], *patchwork* (version 1.3.1) [45], and *mimosa* (version 2.0.0) [42]. CMP graphs were generated for all significant metabolites of the combination group NILM HPV− (n = 8) with HGSIL (n = 11), using the MIMOSA2 contributions results file and the R packages *tidyverse* and *ggplot2*.

## 3. Results

### 3.1. Population Description

Out of the 36 recruited patients, eight (22.22%) were HPV negative and 28 (77.78%) were HPV positive. Of the HPV positive women, 22 (61.11%) were infected with exclusively high-risk HPV types, whereas one (2.78%) patient had a low-risk serotype exclusively. Lastly, five (13.89%) patients were coinfected with both low- and high-risk HPV infections (Table S1). A total of 15 different HPV serotypes were identified within our samples: 12 high-risk and three low-risk (Table S2). The three most prevalent serotypes were high-risk HPV-51, HPV-35, and HPV-16 (Table S2).

### 3.2. Pro-Inflammatory Taxa in Women with High-Severity Cervical Lesions

After quality control assessment, bacterial profiling recovered approximately 566,000 high-quality reads and 2000 ASVs across the four experimental groups: NILM HPV− (n = 8), NILM HPV+ (n = 12), LGSIL (n = 5), and HGSIL (n = 11) (Table 1).

**Figure 1:**
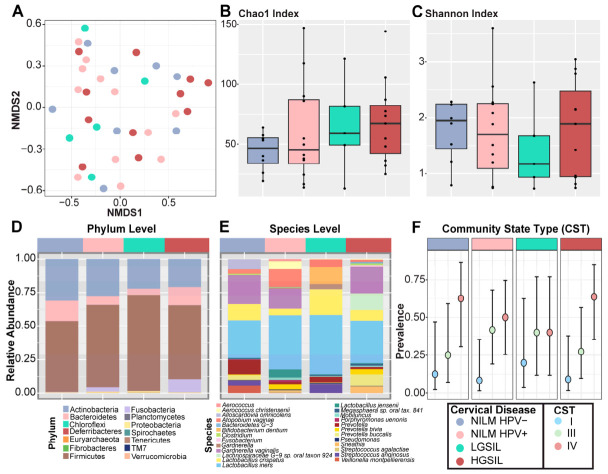
**Higher abundance of inflammatory taxa in women with HGSIL.** No significant differences in microbial structure (**A**), richness (**B**), or diversity (**C**) were observed among cervical disease phenotypes (*p* > 0.05), although HGSIL (n = 11) showed a tendency for the highest richness and diversity overall. At the phylum level (**D**), NILM HPV− patients (n = 8) were enriched in Actinobacteria, whereas NILM HPV+ (n = 12) showed reduced Actinobacteria and Bacteroidetes with increased Firmicutes and Fusobacteria. Patients with low-grade lesions (n = 5) displayed higher Firmicutes and Proteobacteria, while HGSIL samples had the greatest abundance of Fusobacteria. At the species level (**E**), NILM HPV− patients were enriched in *Prevotella*, *Alloscardovia omnicolens*, and *Veillonella montpellierensis*, while NILM HPV+ had higher *Lactobacillus jensenii* and *Atopobium vaginae*. LGSIL had increased *Lactobacillus crispatus* and the highest abundance of *Bifidobacterium dentium* and *Streptococcus anginosus*. Women with HGSIL were characterized by higher levels of *Sneathia*, *Prevotella* spp., *Fusobacterium*, and *Lachnospiraceae G-9 oral taxon 924*. Although no statistical differences were observed, a tendency toward CST IV as the most prevalent across all groups was observed, while CST I was the least abundant (Fisher’s Exact Test *p*-value > 0.05) (**F**).

Prokaryotic community structure and composition according to cervical disease phenotype showed no significant differences as determined by ANOSIM (Figure 1A, Table 2). Alpha diversity metrics were also measured with the Chao1 Index, for the estimated number of species (Figure 1B), and the Shannon Index for diversity measurements (Figure 1C). Although no significant differences were observed in richness or diversity (KW *p*-value > 0.05), patients with high-severity lesions tended to have a higher richness and diversity of the cervicovaginal microbiome. Abundance profiling at the phylum level identified the highest level of Fusobacteria in HGSIL patients and an increase of Proteobacteria in LGSIL patients when compared to the patients with no lesion and no HPV (Figure 1D and Figure S1). Species-level profiling of the top 25 most abundant taxa (Figure 1E) highlighted that the most dominant taxa across the groups was *Lactobacillus iners*, the main species of CST III. Interestingly, several anaerobic bacteria were also observed. Patients with no lesion and no HPV diagnosis had the highest abundance of *Prevotella*, *Alloscardovia omnicolens*, and *Veillonella montpellierensis*, whereas patients with no lesion and a positive HPV diagnosis had a higher abundance of *Lactobacillus jensenii*, *Aerococcus christensenii*, and *Atopobium vaginae*. Women with low- and high-grade lesions showed several distinct bacterial changes. Specifically, LGSIL patients had the highest abundance of *Lactobacillus crispatus*, *Bifidobacterium dentium*, and *Streptococcus anginosus* when compared with all the groups. On the other hand, HGSIL had an increase of *Sneathia*, *Prevotella bivia*, *Prevotella buccalis*, *Fusobacterium*, and *Lachnospiraceae G-9 oral taxon 924*. Although no statistical differences were observed, CST prevalence analysis showed a tendency toward CST IV, the non-*Lactobacillus* dominated state type, as the most prevalent regardless of cervical lesion, whereas CST I was the least abundant (Fisher’s Exact Test *p*-value > 0.05; Table S3; Figure 1F). LGSIL was the only group that showed a tendency for CST III and CST IV to be equally prevalent.

**Figure 2:**
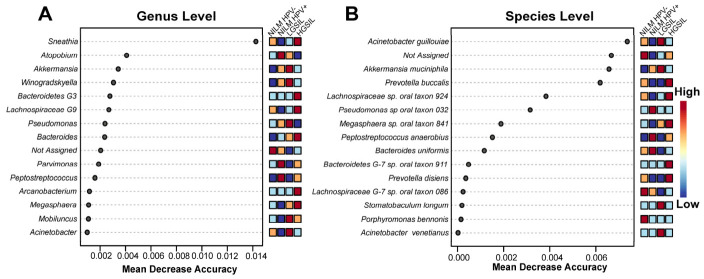
**Exploratory biomarker analysis identifies oral and gut taxa as potential contributors to group discrimination across cervical disease.** Genus-level Random Forest analysis identified *Sneathia* as one of the taxa potentially contributing to group separation in patients with HGSIL (**A**). Species-level analysis showed *Acinetobacter guillouiae* and *Akkermansia muciniphila* among the taxa potentially contributing to the classification of LGSIL patients, whereas *Prevotella buccalis* and *Lachnospiraceae oral taxon 924* for HGSIL (**B**). Analyzed groups included NILM HPV− (n = 8), NILM HPV+ (n = 12), LGSIL (n = 5), and HGSIL (n = 11).

Subsequently, as an exploratory approach, Random Forest analysis was used to identify bacterial taxa contributing to group discrimination. Due to the small sample size, the Random Forest model yielded a relatively high OOB error, suggesting limited classification performance. Hence, these results should not be considered validated biomarkers and should be regarded only as potential contributors to group discrimination (Table S4). At the genus level, *Sneathia* was identified as a potential contributor to the classification of HGSIL patients (Figure 2A, Table S4). Species-level analysis identified *Acinetobacter guillouiae* and *Akkermansia muciniphila* as potential classification contributors for women with low-grade lesions, whereas patients with more severe cervical lesions showcased *Prevotella buccalis* and *Lachnospiraceae G-9 oral taxon 924* (Figure 2B, Table S4).

### 3.3. Biogenic Amines Are More Abundant in Cervical Lesion Severity

**Figure 3:**
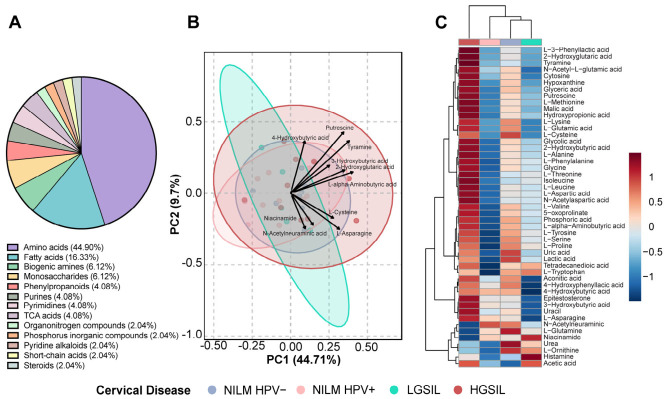
**Differential abundance of metabolites across cervical disease phenotypes highlights biogenic amines in HGSIL patients**. Pie chart depicting the metabolites’ main classes (**A**), where amino acids constituted 44.90% of identified metabolites. No significant differences were observed in metabolite presence across the cervical phenotypes, although a nominal significance between NILM HPV+ and HGSIL patients was seen (PERMANOVA *p*-value = 0.049), but did not remain after multiple testing correction (PERMANOVA adjusted *p*-value = 0.294) (**B**). HGSIL patients showcased a tendency toward a higher abundance of most metabolites–specifically tyramine and putrescine (ANOVA and Tukey’s HSD *p*-value > 0.05) (**C**). Analyzed groups included NILM HPV− (n = 8), NILM HPV+ (n = 12), LGSIL (n = 5), and HGSIL (n = 11). The pie chart percentages have been rounded to two decimal places.

Untargeted metabolomics identified 49 unique metabolites, which were classified according to their main class (Figure 3A). The metabolic features were mainly classified as amino acids (44.90%), followed by fatty acids (16.33%). The third most abundant classification was biogenic amines and monosaccharides, both at 6.12%. Phenylpropanoids, purines, pyrimidines, and TCA acids occupied fourth place with 4.08% each. Lastly, organonitrogen compounds, phosphorous inorganic compounds, pyridine alkaloids, short-chain acids, and steroids occupied fifth place with 2.04% each. Overall, metabolite profiles did not differ significantly across cervical lesions (Figure 3B, Table S5). Although the NILM HPV+ versus HGSIL comparison showed nominal significance between patients (PERMANOVA *p*-value = 0.049), after multiple testing correction, it did not remain significant (PERMANOVA adjusted *p*-value = 0.294) (Figure 3B, Table S5). In addition, a PCA biplot shows arrows with potential metabolites responsible for differences between groups. Specifically, putrescine and tyramine showed a directional trend toward the HGSIL samples, suggesting a possible increase in biogenic amine metabolism in higher-grade lesions (Figure 3B). Although no statistical significance was observed (ANOVA and Tukey’s HSD *p*-value > 0.05), differential abundance analysis highlighted a trend in metabolic shifts by cervical disease severity (Figure 3C, Tables S6 and S7). Specifically, putrescine and tyramine tended to higher abundance in a subset of patients with the highest severity of cervical lesions (Figure 3C).

### 3.4. Abundances of Biogenic Amine Metabolites Are Associated with Microbial Metabolic Potential in the Cervix

**Figure 4:**
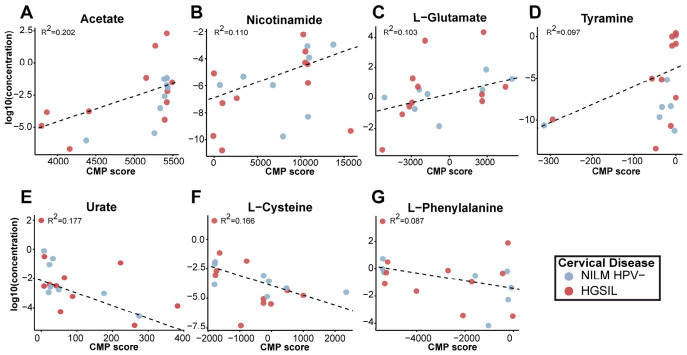
**Predicted metabolites identified by the community metabolic potential (CMP) model.** Log-transformed concentrations alongside the community metabolic potential were visualized for significant metabolites with positive (**A**–**D**) and negative (**E**–**G**) slopes. Analyzed groups included NILM HPV− (n = 8) and HGSIL (n = 11).

To evaluate and relate variations in metabolite measurements to microbial composition using the KEGG database, we used the MIMOSA2 platform. This tool identifies metabolites whose abundances are consistent with microbiome metabolic capabilities by comparing community metabolic potential (CMP) scores with metabolite abundances. CMP scores are based on the inferred abundance of microbial genes linked to transformations of a given metabolite. We defined consistent metabolites as those with a model *p*-value < 0.1 and potential contributing taxa and reactions as those that explained greater than 1% of variance in metabolite levels according to the model. We integrated bacteriome and metabolome data into the sub-grouping of NILM HPV− (n = 8) and HGSIL (n = 11) patients. Abundances of several metabolites were significantly associated with predicted microbial metabolic potential in the bacterial communities (Figure 4). Metabolites significantly associated with CMP scores between NILM HPV− and HGSIL patients, including acetate (Figure 4A), nicotinamide (Figure 4B), L-glutamate (Figure 4C), tyramine (Figure 4D), urate (Figure 4E), L-cysteine (Figure 4F), and L-phenylalanine (Figure 4G).

**Figure 5:**
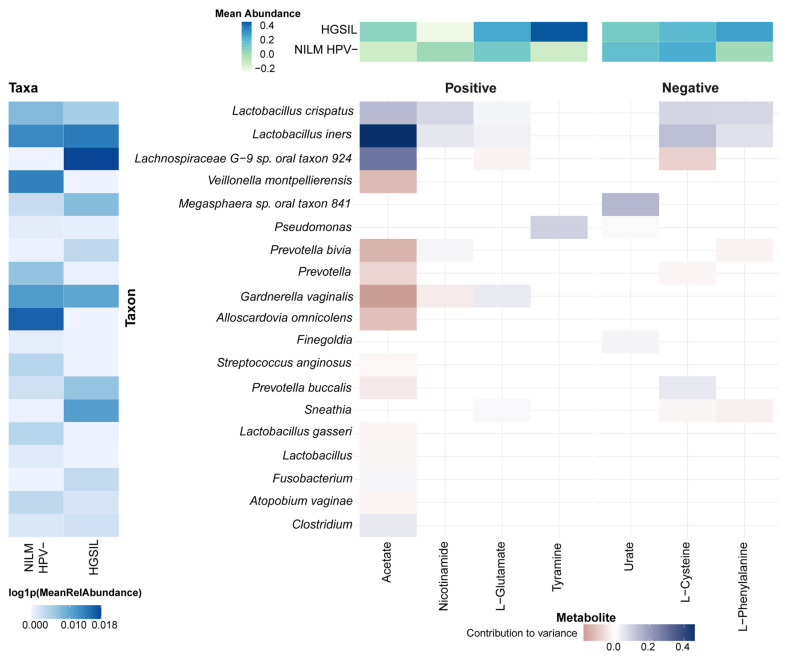
**Diverse taxa explain variation in biogenic amines and other metabolites across HPV infection status.** Concentration abundance of the biogenic amine tyramine across cervical phenotypes shows that patients with high-severity lesions had the highest abundance. Tyramine was identified as an important differential metabolite across groups (*p*-value < 0.1; VarShare ± 0.01). Differences in the relative capacity of community members to degrade tyramine identified by MIMOSA2 showed *Pseudomonas* as the highest potential contributor, although the relative mean abundance of this taxon is similar across both cervical disease phenotypes. Analyzed groups included NILM HPV− (n = 8) and HGSIL (n = 11).

Several oral taxa, such as *Lachnospiraceae G-9 oral taxon 924*, *Veillonella montpellierensis*, *Megasphaera* sp. *oral taxon 841*, *Prevotella bivia*, and *Prevotella buccalis*, were linked to the synthesis or degradation of metabolites within the cervix (Figure 5). *Lactobacillus iners* and *Lachnospiraceae G-9 oral taxon 924* were predicted as strong contributors for the synthesis of acetate, among other community members, on the basis of the following genes: (1) lactate 2-monooxygenase (K00467), (2) acylphosphatase (K01512), (3) cysteine synthase (K01738), and (4) pyruvate dehydrogenase (K00156). Nicotinamide synthesis was linked to *Lactobacillus iners*, *Lactobacillus crispatus*, and *Gardnerella vaginalis* through purine nucleosidase (K01239), whereas *Prevotella bivia* was the sole taxon linked to degradation of nicotinamide via nicotinamidase/pyrazinamidase (K08281). While many taxa were identified as potential synthesizers and degraders of L-glutamate (*Lactobacillus iners*, *Lactobacillus crispatus*, *Lachnospiraceae G-9 oral taxon 924*, and *Gardnerella vaginalis*), *Sneathia* was predicted to only have degradation enzymes, specifically glutamyl-tRNA synthetase (K01885).

**Figure 6:**
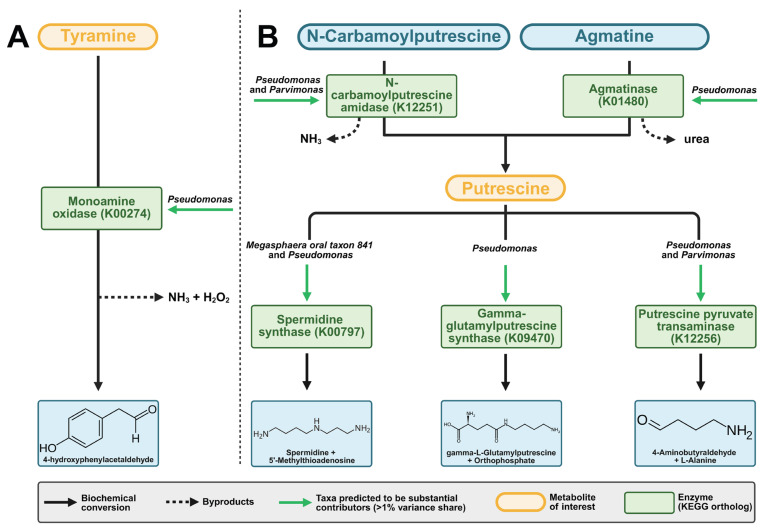
**Hypothesized model of biogenic amine degradation by the cervicovaginal microbiome**. Pathways reconstructed based on KEGG orthologs depicting taxon-specific function attributions to tyramine (**A**) and putrescine-related metabolism (**B**). The green arrow indicates taxa that were predicted by MIMOSA2 to be substantial contributors to the corresponding function (>1% share of variation). Created in BioRender under an institutional academic license by Meléndez-Vázquez, N.M. (2026). https://BioRender.com/a7uiyf5 (accessed on 10 January 2026).

Lastly, the biogenic amine identified as an important differential metabolite across our groups was tyramine. Interestingly, the only taxon predicted to explain variation in tyramine abundances was *Pseudomonas*, via the degradation enzyme monoamine oxidase (K00274) (Figure 5). This tyramine degradation pathway results in the production of 4-hydroxyphenylacetaldehyde alongside the byproducts ammine (NH_3_) and hydrogen peroxide (H_2_O_2_) (Figure 6A). Although *Pseudomonas* was roughly equally abundant across the groups, tyramine was more abundant in HGSIL patients than in the non-lesion non-HPV patients, suggesting that other contributing factors (microbial or otherwise) likely influence its abundance. Additionally, three other metabolites (urate, L-cysteine, and L-phenylalanine) were identified as significantly negatively associated with CMP scores in the control patients with no HPV/no lesion and the HGSIL women. All identified taxa for urate were potential synthesizers of this metabolite, with *Megasphaera oral taxon 841* explaining the largest share of variation through the enzyme xanthine dehydrogenase (K13479) (Figure 5). For L-cysteine, we observed *Lactobacillus crispatus*, *Lactobacillus iners*, *Lachnospiraceae G-9 oral taxon 924*, and *Prevotella buccalis* as the main contributors, all harboring co-occurring genes for production and degradation of the metabolite. Lastly, for L-phenylalanine, only predicted genes linked to degradation accounted for a substantial share of variation (phenylalanyl-tRNA synthetase alpha chain (K01889) and beta chain (K01890)).

To further understand potential microbial and metabolomic differences across cervical dysplasia severity, we performed an integration analysis for a sub-cohort comparing low- (n = 5) and high-grade (n = 11) cervical lesions. This analysis revealed predicted contributions of genes linked to both synthesis and degradation of putrescine from oral taxa such as *Megasphaera oral taxon 841*, *Pseudomonas*, and *Parvimonas* (Figure S2 and Figure 6B). Additionally, other oral taxa, such as *Bifidobacterium dentium*, were linked to the potential synthesis of acetate (K01738) and nicotinamide (K01239). *Bifidobacterium dentium* was also linked to both potential synthesis (K00265 and K00266) and degradation (K01885, K01915, and K01919) of L-glutamate (Figure S2). To assess whether BMI affected the identification of contributors, we performed a sub-cohort analysis of only normal-weight patients in the negative controls (n = 6) and HGSIL (n = 4) groups (Figure S3). Here, we observed the same trend in tyramine degradation mediated by *Pseudomonas* and linked to the enzyme monoamine oxidase (K00274). Lastly, we performed an additional sub-cohort analysis of patients with the most severe cervical lesions (HGSIL) and compared between normal-weight (n = 4) and overweight/obese (n = 6) classifications (Figure S4). As in the previous comparisons, *Pseudomonas* was linked to tyramine degradation, and we also observed how this taxon was a potential contributor to urea degradation via the urease enzyme (K01428, K01429, and K01430) (Figure S4). Overall, taxa commonly found in the oral cavity remained the predictors of metabolic differences across cervical dysplasia.

## 4. Discussion

This is the first study to assess the integration of bacterial communities with metabolomic shifts in the cervicovaginal tract of women living in Puerto Rico. Similar to previous studies, our cohort of reproductive-age non-pregnant Puerto Rican women revealed CST III, dominated by *Lactobacillus iners*, and CST IV, the diverse anaerobic group, as the most abundant community types [5]. These results highlight how Puerto Ricans withstand a high inflammatory milieu resonating with a dysbiotic state as their baseline. Within our cohort, we encountered a higher prevalence of high-risk HPV serotypes, with the most dominant HPV-51, followed by HPV-35 and HPV-16, with few HPV-18. This seems to shed light on the limited coverage provided by the quadrivalent and 9-valent HPV vaccines, which do not protect against HPV-51 and HPV-35. Additionally, the HPV rate observed in this study cohort (28/36 = 77.78%) is consistent with previous findings, including overall HPV prevalence of 74.8% [5] and 84% [16].

Pairing metabolomics with amplicon sequencing offers a unique platform to assess the complex interactions between microbially-produced metabolites and HPV in the cervicovaginal niche. This approach allowed us to identify microbes that may enhance a pro-inflammatory state in the cervix, ideal for HPV persistence and cervical dysplasia progression. Specifically, bacterial community analyses showed an increased prevalence of pro-inflammatory taxa, such as *Prevotella bivia*, *Sneathia*, and *Fusobacterium*, in patients suffering lesions of higher severity. Anaerobes, such as *Prevotella* and *Sneathia*, have been associated with inducing the recruitment of Th17 lymphocytes which cause inflammation in the vaginal tract [46]. *Prevotella* has also been linked to providing nutrients to other vaginal bacteria. For example, ammonia produced by *Prevotella bivia* supports the growth of *Gardnerella vaginalis*, providing evidence of a symbiotic relationship between both taxa [47]. Thus, this may promote an environment that could influence HPV persistence and cervical cancer [48]. Integration approaches identified *Prevotella bivia* as a key contributor for nicotinamide degradation, although little is understood as to how this could potentially affect cervical disease progression. Like previous studies, *Sneathia* was found in more abundance in HPV positive patients with cervical lesions, highlighting its potential role as a disease severity biomarker [49,50,51,52]. Furthermore, *Sneathia* has been associated with inducing pro-inflammatory cytokines, such as IL-1β, thereby creating a microenvironment ideal for HPV infection [18]. *Fusobacterium*, on the other hand, has been linked with carcinogenesis through chronic inflammation. Cervical cancer patients with a high burden of *Fusobacterium* intratumorally have exhibited lower overall survival, highlighting its potential as a diagnostic biomarker [53].

Interestingly, patients with cervical dysplasia showed more prevalence of taxa commonly found in the oral cavity, including *Prevotella buccalis*, *Lachnospiraceae G-9 oral taxon 924*, *Bifidobacterium dentium*, and *Streptococcus anginosus*. Specifically, *Prevotella buccalis* and *Streptococcus anginosus* are oral commensals that have been associated with periodontal disease and dental plaque [54,55], while *Bifidobacterium dentium* has been detected in caries [56]. In microbial–metabolome integrations comparing LGSIL and HGSIL patients, *Megasphaera oral taxon 841* and *Parvimonas* were identified as potential putrescine degraders, forming spermidine and 4-aminobutyraldehyde with L-alanine, respectively. Additionally, *Pseudomonas* had a dual role in the synthesis and degradation of putrescine. In a similar manner, comparisons between HPV negative patients with no cervical lesions and HGSIL patients highlighted *Pseudomonas* as a potential contributor to tyramine degradation. However, the relative abundance of *Pseudomonas* remained similar across both groups, suggesting that tyramine accumulation within the HGSIL group may reflect increased synthesis by other microbial community members or host-related metabolic pathways. Specifically, MIMOSA2 accounts for only a limited number of well-characterized metabolic reactions; hence, it is plausible that other microbial transformations involving tyramine metabolism occur and are not fully captured in the model. Given the heterogeneity of microbial communities across subjects, different taxa can contribute to tyramine metabolism in different individuals, highlighting that multiple factors likely impact tyramine levels. Hence, even though there is an overall correlation between tyramine and *Pseudomonas*, other microbes may have a larger impact in some HGSIL samples (not captured by MIMOSA2). Importantly, although *Pseudomonas* is not considered a dominant member of the cervicovaginal microbiome, several studies have previously associated this species with vaginal dysbiosis, HPV-associated cervical disease, and cervical cancer [57,58,59,60,61]. However, negative controls were not included for the 16S rRNA sequencing workflow in this study. This limits the ability to fully exclude environmental or processing contamination, which is particularly relevant for low-abundance taxa such as *Pseudomonas*. Therefore, *Pseudomonas* detection and predicted contribution to tyramine metabolism should be validated in future studies. Previously, researchers have found that HPV+ women had a higher prevalence of biogenic amines compared to HPV− patients [23]. These polyamines disrupt Lactobacilli within the cervicovaginal environment, contributing to a dysbiotic state consistent with that of bacterial vaginosis [23]. These results highlight the potential role that sexual behaviors, such as oral sex, may contribute to the translocation of microbes that may contribute to cervical disease. Hence, it is important to further evaluate the impact that sexual practices may contribute toward HPV persistence and disease progression.

While our results shed light on complex host–microbe interactions within the cervicovaginal tract, our study holds several limitations. First, the relatively small sample size, particularly within the LGSIL group, may limit statistical power, reducing the ability to detect subtle but biologically meaningful differences across cervical disease phenotypes. This constraint also limits the generalizability of the results to broader populations. Hence, the findings should be interpreted with caution and validated in larger longitudinal cohorts. Second, we integrated our microbiome and metabolome data using MIMOSA2, which hypothesizes potential mechanistic links between microbes and metabolites based on evidence of quantitative associations and the presence (or inferred presence) of functionally relevant genes. However, it does not capture host metabolism, signaling processes, or transcriptional regulation [6]. Similarly, the tool assigns these associations only to irreversible reactions, while information from reversible reactions is lost, hindering the prediction of other metabolite pathways [6]. Although the MIMOSA2 model identified specific taxa as associated with tyramine abundance and capable of tyramine degradation, these predictions are based on computational inference. Therefore, functional conclusions about microbial metabolic activity remain tentative and require validation through experimental assays. This cross-sectional design precludes any inference of causality between microbiome composition, predicted metabolic activity, and disease progression. Likewise, the absence of negative controls in 16S rRNA sequencing limits the ability to fully exclude potential contamination. Finally, potential confounding factors such as host genetics, hormonal status, diet, and other environmental influences were not fully controlled and may have influenced microbial composition and metabolic predictions.

We believe that future studies with larger, longitudinal cohorts, implementation of better techniques for broad-spectrum metabolite detection, such as Liquid Chromatography–Mass Spectrometry [62], and integrated multi-omics approaches are needed to validate these findings and better elucidate the role of microbiome-derived metabolites in cervical disease progression.

## 5. Conclusions

Despite the low sample size, this study provides new insights into microbial and metabolic pathways that may contribute to HPV persistence and cervical dysplasia in women living in Puerto Rico. These results suggest that strictly anaerobic bacteria and oral-associated taxa in the cervicovaginal tract may influence disease processes through polyamine-related pathways, potentially contributing to cancer invasion. Further studies are warranted to unravel the molecular mechanisms underlying cervical disease progression.

## Figures and Tables

**Table 1:** Patient cohort description by cervical disease phenotype and bacterial sequence summarization.

Cervical Disease Phenotype	Number of Samples	Average Reads ± Stdev ^1^	Average ASVs ± Stdev ^1^
NILM (HPV−)	8	17,026.63 ± 8260.98	44.88 ± 13.53
NILM (HPV+)	12	15,241.75 ± 4148.47	61.67 ± 36.43
LGSIL (HPV+)	5	12,638.20 ± 5236.78	55.40 ± 34.83
HGSIL (HPV+)	11	16,747.73 ± 7110.46	87.36 ± 28.37
**Grand total**	**36**		

^1^ Stdev = standard deviation. Bold formatting is used to denote the grand total row and sample size of the analyzed cohort.

**Table 2:** Pairwise statistical tests of cervical disease phenotype analysis.

		Figure 1A	Figure 1B	Figure 1C
Experimental Group 1	Experimental Group 2	ANOSIM *p*-Value	Chao1 KW *p*-Value	Shannon KW *p*-Value
NILM (HPV−)	NILM (HPV+)	0.576	0.700	0.589
NILM (HPV−)	LGSIL	0.875	0.306	0.306
NILM (HPV−)	HGSIL	0.725	0.117	0.869
NILM (HPV+)	LGSIL	0.649	0.752	0.461
NILM (HPV+)	HGSIL	0.404	0.538	0.758
LGSIL	HGSIL	0.356	0.955	0.396

## Data Availability

16S rRNA gene sequences can be found in the QIITA study 12871, sandbox ID 7969 (https://qiita.ucsd.edu/study/description/12871, accessed on 10 January 2025). They are also available in the European Nucleotide Archive Project (PRJEB51893, ERP136546).

## Supplementary Materials

The following supporting information can be downloaded at: https://www.mdpi.com/article/10.3390/cancers18121931/s1, Figure S1: Exploratory phylum-level Random Forest analysis of patients with cervical disease. Fusobacteria was identified as a potential contributor to the HGSIL group classification, while Proteobacteria was observed for the LGSIL group.; Figure S2: Diverse taxa explain biogenic amine variation across cervical dysplasia progression. Concentration abundance of the biogenic amine putrescine across cervical phenotypes shows that patients with HGSIL had the highest abundance when compared with patients with low-grade lesions. Putrescine was identified as an important differential metabolite across groups (*p*-value < 0.1; VarShare ± 0.01). Differences in the relative capacity of community members to synthesize putrescine identified by MIMOSA2 showed *Pseudomonas* and *Parvimonas* as the highest contributors, whereas degradation of putrescine was driven by *Pseudomonas*, *Parvimonas*, and *Megasphaera oral taxon 841*. Analyzed groups included LGSIL (n = 5) and HGSIL (n = 11).; Figure S3: Tyramine degradation remains a significant pathway in cervical dysplasia regardless of BMI. Analyzed groups included normal weight patients classified as NILM HPV− (n = 6) and HGSIL (n = 4).; Figure S4: Tyramine degradation is a significant pathway when comparing normal and overweight/obese HGSIL patients. *Pseudomonas* was associated with tyramine and urea metabolism when comparing patients with high-severity lesions across BMI classifications. Taxa commonly found in the oral cavity remained as potential contributors of metabolic differences across the groups. Analyzed groups included HGSIL patients classified as normal weight (n = 4) and obese/overweight (n = 6).; Table S1: Number of patients with cervical HPV risk types; Table S2: Distribution of HPV serotype detection according to HPV risk classification; Table S3: Fisher’s Exact Test pairwise statistics for CST prevalence; Table S4: Random forest (OOB error) pairwise statistical test; Table S5: PERMANOVA pairwise statistical test for metabolite PCA biplot; Table S6: ANOVA with multiple hypothesis correction for untargeted metabolites; Table S7: Pairwise Tukey’s HSD test for untargeted metabolites.

## Abbreviations

The following abbreviations are used in this manuscript:

ANOSIM	Analysis of Similarities
ANOVA	Analysis of Variance
AMDIS	Automated Mass Spectral Deconvolution and Identification System
ASV	Amplicon Sequence Variant
BMI	Body Mass Index
bp	Base-Pairs
CMP	Community-Level Metabolic Potential
CST	Community State Type
CVL	Cervical Lavage
EI	Electron Impact Ionization
ENA	European Nucleotide Archive Project
eV	Electronvolt
GC-MS	Gas Chromatography-Mass Spectrometry
gDNA	Genomic Deoxyribonucleic Acid
HGSIL	High-Grade Squamous Intraepithelial Lesion
HIPAA	Health Insurance Portability and Accountability Act
HPV	Human Papillomavirus
HPV -	HPV negative
HPV +	HPV positive
HSD	Honestly Significant Difference
IRB	Institutional Review Board
kDA	Kilodaltons
KEGG	Kyoto Encyclopedia of Genes and Genomes
KW	Kruskal–Wallis
LGSIL	Low-Grade Squamous Intraepithelial Lesion
MIMOSA2	Model-Based Integration of Metabolite Observations and Species Abundances 2
mL	Milliliters
mM	Millimolar
MSTFA	N-methyl-N-trimethylsilyl-trifluoroacetamide
m/z	Mass-to-Charge Ratio
NILM	Negative For Intraepithelial Lesion or Malignancy
NMDS	Non-Metric Multidimensional Scaling
OOB	Out-Of-Bag
PCA	Principal Component Analysis
PERMANOVA	Permutational Multivariate Analysis of Variance
PICRUSt2	Phylogenetic Investigation of Communities by Reconstruction of Unobserved States
rRNA	Ribosomal Ribonucleic Acid
Stdev	Standard Deviation
STIs	Sexually Transmitted Diseases
Th	T-helper
TMCS	Trimethylchlorosilane
UPR	University of Puerto Rico
VALENCIA	Vaginal Community State Type Nearest Centroid Classifier
VarShare	Variance Share
°C	Degree Celsius
