# Supplementary material for: Cervicovaginal Microbiota and Biogenic Amine Metabolic Shifts in HPV-Associated Cervical Disease
Source: Cancers (Basel). 2026 Jun 13;18(12):1931. doi: 10.3390/cancers18121931 (PMC13296784; doi:10.3390/cancers18121931)
Supplement: Supplementary file 1 [file cancers-18-01931-s001.zip › cancers-4276060-supplementary.pdf]

Table S1: Number of patients with cervical HPV risk types.

HPV Status	HPV Risk Type	Number of samples
**HPV negative**	-	8
**HPV positive**	Low-risk HPV	1
	High-risk HPV	22
	Both Low- and High-risk HPV	5
		**36**

Table S2: Distribution of HPV serotype detection according to HPV risk classification.

	HPV Risk Type	HPV Serotype	HPV Prevalence	Subtotal
**SPF10-LiPA Assay**	**Low-risk HPV**	44	1	**5 (10%)**
		53	1	
		74	3	
	**High-risk HPV**	16	6	**45 (90%)**
		18	2	
		31	2	
		33	4	
		35	7	
		39	1	
		45	2	
		51	10	
		52	4	
		56	5	
		58	1	
		68	1	
				**50 (100%)**

Table S3: Fisher's Exact Test pairwise statistics for CST prevalence.

CST	Group 1	Group 2	Success Group 1	Total Group 1	Success Group 2	Total Group 2	*p*-value	Odds Ratio
I	NILM HPV−	NILM HPV+	1	8	1	12	1.000	1.5353
I	NILM HPV−	LGSIL	1	8	1	5	1.000	0.5976
I	NILM HPV−	HGSIL	1	8	1	11	1.000	1.4015
I	NILM HPV+	LGSIL	1	12	1	5	0.515	0.3892
I	NILM HPV+	HGSIL	1	12	1	11	1.000	0.9129
I	LGSIL	HGSIL	1	5	1	11	1.000	2.3452
III	NILM HPV−	NILM HPV+	2	8	5	12	0.642	0.4846
III	NILM HPV−	LGSIL	2	8	2	5	1.000	0.5286
III	NILM HPV−	HGSIL	2	8	3	11	1.000	0.8944
III	NILM HPV+	LGSIL	5	12	2	5	1.000	1.0671
III	NILM HPV+	HGSIL	5	12	3	11	0.667	1.8518
III	LGSIL	HGSIL	2	5	3	11	1.000	1.7120
IV	NILM HPV−	NILM HPV+	5	8	6	12	0.670	1.6245
IV	NILM HPV−	LGSIL	5	8	2	5	0.592	2.3236
IV	NILM HPV−	HGSIL	5	8	7	11	1.000	0.9549
IV	NILM HPV+	LGSIL	6	12	2	5	1.000	1.4647
IV	NILM HPV+	HGSIL	6	12	7	11	0.680	0.5856
IV	LGSIL	HGSIL	2	5	7	11	0.596	0.4058

Table S4: Random forest (OOB error) pairwise statistical test.

GENUS LEVEL (Figure 2A)						
	**NILM HPV−**	**NILM HPV+**	**LGSIL**	**HGSIL**	**Class Error**	**OOB Error**
**NILM HPV−**	1.0	4.0	0.0	3.0	0.875	0.694
**NILM HPV+**	2.0	6.0	1.0	3.0	0.500	
**LGSIL**	1.0	4.0	0.0	0.0	1.000	
**HGSIL**	2.0	5.0	0.0	4.0	0.636	
**SPECIES LEVEL (Figure 2B)**						
	**NILM HPV−**	**NILM HPV+**	**LGSIL**	**HGSIL**	**Class Error**	**OOB Error**
**NILM HPV−**	1.0	4.0	0.0	3.0	0.875	0.778
**NILM HPV+**	2.0	5.0	2.0	3.0	0.583	
**LGSIL**	1.0	3.0	0.0	1.0	1.000	
**HGSIL**	4.0	5.0	0.0	2.0	0.818	
**PHYLUM LEVEL (Figure S1)**						
	**NILM HPV−**	**NILM HPV+**	**LGSIL**	**HGSIL**	**Class Error**	**OOB Error**
**NILM HPV−**	2.0	3.0	0.0	3.0	0.750	0.667
**NILM HPV+**	1.0	6.0	2.0	3.0	0.500	
**LGSIL**	1.0	4.0	0.0	0.0	1.000	
**HGSIL**	2.0	5.0	0.0	4.0	0.636	

Table S5: PERMANOVA pairwise statistical test for metabolite PCA biplot.

PERMANOVA (Figure 3B)					
**Group 1**	**Group 2**	**F.Model**	**R2**	***p*-value**	***p*-adjusted**
NILM HPV−	NILM HPV+	1.41830	0.07304	0.25700	0.54000
NILM HPV−	LGSIL	0.45297	0.03955	0.62000	0.74400
NILM HPV−	HGSIL	0.68193	0.03857	0.46300	0.69450
NILM HPV+	LGSIL	0.10648	0.00705	0.87800	0.87800
NILM HPV+	HGSIL	3.49620	0.14272	0.04900	0.29400
LGSIL	HGSIL	1.24930	0.08193	0.27000	0.54000

Table S6: ANOVA with multiple hypothesis correction for untargeted metabolites.

ANOVA (Figure 3C)		
**Metabolite Name**	***p*-value**	***p*-adjusted**
2-Hydroxybutyric acid	0.232587661	0.579586969
2-Hydroxyglutaric acid	0.050794426	0.578862257
3-Hydroxybutyric acid	0.077472911	0.578862257
4-Hydroxybutyric acid	0.737628511	0.816946024
4-Hydroxyphenyllactic acid	0.354849165	0.579586969
5-oxoprolinate	0.383273136	0.58688699
Acetic acid	0.962857698	0.962857698
Aconitic acid	0.352850531	0.579586969
Cytosine	0.766928921	0.816946024
Epitestosterone	0.335964417	0.579586969
Glyceric acid	0.070823041	0.578862257
Glycine	0.466682095	0.633037836
Glycolic acid	0.30617201	0.579586969
Histamine	0.536621591	0.69195942
Hydroxypropionic acid	0.110773811	0.578862257
Hypoxanthine	0.140999586	0.578862257
Isoleucine	0.185221891	0.579586969
L-3-Phenyllactic acid	0.233766131	0.579586969
L-Alanine	0.075838725	0.578862257
L-Asparagine	0.321991211	0.579586969
L-Aspartic acid	0.274003023	0.579586969
L-Cysteine	0.601956267	0.741806472
L-Glutamic acid	0.43051416	0.614091917
L-Glutamine	0.734532563	0.816946024
L-Leucine	0.345514609	0.579586969
L-Lysine	0.378434958	0.58688699
L-Methionine	0.156933675	0.578862257
L-Ornithine	0.434317982	0.614091917
L-Phenylalanine	0.438637084	0.614091917
L-Proline	0.793690933	0.827465015

L-Serine	0.322805843	0.579586969
L-Threonine	0.128078667	0.578862257
L-Tryptophan	0.291747876	0.579586969
L-Tyrosine	0.329122034	0.579586969
L-Valine	0.130636401	0.578862257
L-alpha-Aminobutyric acid	0.620695211	0.741806472
Lactic acid	0.478008162	0.633037836
Malic acid	0.015886455	0.578862257
N-Acetyl-L-glutamic acid	0.100707867	0.578862257
N-Acetylaspartic acid	0.124766728	0.578862257
N-Acetylneuraminic acid	0.163270233	0.578862257
Niacinamide	0.879135331	0.89745065
Phosphoric acid	0.319686238	0.579586969
Putrescine	0.290499047	0.579586969
Tetradecanedioic acid	0.295843134	0.579586969
Tyramine	0.616948749	0.741806472
Uracil	0.165389216	0.578862257
Urea	0.762235007	0.816946024
Uric acid	0.684895138	0.799044328

Table S7: Pairwise Tukey's HSD test for untargeted metabolites.

Tukey HSD (Figure 3C)							
**Metabolite Name**	**Group 1**	**Group 2**	**Null Value**	**Estimate**	**Conf. Low**	**Conf. High**	***p* adjusted**
2-Hydroxybutyric acid	NILM HPV−	NILM HPV+	0	-0.368486712	-1.370996548	0.634023125	0.753
	NILM HPV−	LGSIL	0	-0.145228144	-1.397362528	1.106906241	0.989
	NILM HPV−	HGSIL	0	0.34135132	-0.67922321	1.36192585	0.802
	NILM HPV+	LGSIL	0	0.223258568	-0.945858758	1.392375894	0.954
	NILM HPV+	HGSIL	0	0.709838032	-0.206986475	1.626662539	0.176
	LGSIL	HGSIL	0	0.486579464	-0.698064669	1.671223597	0.684
2-Hydroxyglutaric acid	NILM HPV−	NILM HPV+	0	-0.295180996	-1.347622895	0.757260904	0.872
	NILM HPV−	LGSIL	0	-0.31493399	-1.629433501	0.999565522	0.915
	NILM HPV−	HGSIL	0	0.664996308	-0.406410035	1.73640265	0.35
	NILM HPV+	LGSIL	0	-0.019752994	-1.247100612	1.207594624	1
	NILM HPV+	HGSIL	0	0.960177303	-0.002311533	1.92266614	0.0507
	LGSIL	HGSIL	0	0.979930297	-0.263717472	2.223578067	0.164
3-Hydroxybutyric acid	NILM HPV−	NILM HPV+	0	0.071120146	-1.227049213	1.369289505	0.999
	NILM HPV−	LGSIL	0	-0.711190974	-2.332603984	0.910222036	0.638
	NILM HPV−	HGSIL	0	0.774859538	-0.546702142	2.096421218	0.399
	NILM HPV+	LGSIL	0	-0.78231112	-2.296223737	0.731601497	0.509
	NILM HPV+	HGSIL	0	0.703739392	-0.483474378	1.890953162	0.39
	LGSIL	HGSIL	0	1.486050512	-0.047968067	3.020069091	0.0604
4-Hydroxybutyric acid	NILM HPV−	NILM HPV+	0	-0.025885987	-1.699235962	1.647463988	1
	NILM HPV−	LGSIL	0	-0.698844109	-2.788857558	1.39116934	0.802
	NILM HPV−	HGSIL	0	0.091564609	-1.611938242	1.795067459	0.999

	NILM HPV+	LGSIL	0	-0.672958122	-2.624402764	1.278486519	0.787
	NILM HPV+	HGSIL	0	0.117450596	-1.412876799	1.64777799	0.997
	LGSIL	HGSIL	0	0.790408718	-1.186952659	2.767770094	0.702
4-Hydroxyphenyllactic acid	NILM HPV−	NILM HPV+	0	-0.340776582	-1.789551235	1.107998072	0.919
	NILM HPV−	LGSIL	0	-1.047412051	-2.856931013	0.762106911	0.41
	NILM HPV−	HGSIL	0	0.026422435	-1.448458367	1.501303236	1
	NILM HPV+	LGSIL	0	-0.706635469	-2.396182532	0.982911593	0.672
	NILM HPV+	HGSIL	0	0.367199016	-0.957747692	1.692145724	0.876
	LGSIL	HGSIL	0	1.073834486	-0.638151104	2.785820075	0.341
5-oxoprolinate	NILM HPV−	NILM HPV+	0	-0.385039319	-1.41398686	0.643908221	0.743
	NILM HPV−	LGSIL	0	-0.106354659	-1.391509725	1.178800407	0.996
	NILM HPV−	HGSIL	0	0.223560344	-0.823928284	1.271048971	0.938
	NILM HPV+	LGSIL	0	0.278684661	-0.921264062	1.478633383	0.922
	NILM HPV+	HGSIL	0	0.608599663	-0.332402896	1.549602222	0.314
	LGSIL	HGSIL	0	0.329915003	-0.885969992	1.545799997	0.882
Acetic acid	NILM HPV−	NILM HPV+	0	0.062915752	-1.123746031	1.249577534	0.999
	NILM HPV−	LGSIL	0	0.203990321	-1.27814977	1.686130412	0.982
	NILM HPV−	HGSIL	0	0.201385959	-1.006658837	1.409430754	0.969
	NILM HPV+	LGSIL	0	0.14107457	-1.242798984	1.524948123	0.992
	NILM HPV+	HGSIL	0	0.138470207	-0.946766629	1.223707044	0.986
	LGSIL	HGSIL	0	-0.002604362	-1.404856856	1.399648131	1
Aconitic acid	NILM HPV−	NILM HPV+	0	-0.349998468	-1.687143331	0.987146396	0.893
	NILM HPV−	LGSIL	0	-0.844434573	-2.514527972	0.825658826	0.527
	NILM HPV−	HGSIL	0	0.142339547	-1.218899955	1.503579049	0.992

	NILM HPV+	LGSIL	0	-0.494436105	-2.05380158	1.06492937	0.826
	NILM HPV+	HGSIL	0	0.492338015	-0.730519992	1.715196021	0.698
	LGSIL	HGSIL	0	0.98677412	-0.593300967	2.566849206	0.344
Cytosine	NILM HPV−	NILM HPV+	0	-0.187544667	-1.668966122	1.293876788	0.986
	NILM HPV−	LGSIL	0	-0.298512119	-2.148806923	1.551782685	0.972
	NILM HPV−	HGSIL	0	0.26841843	-1.239697451	1.77653431	0.962
	NILM HPV+	LGSIL	0	-0.110967452	-1.8385869	1.616651995	0.998
	NILM HPV+	HGSIL	0	0.455963097	-0.898840064	1.810766257	0.799
	LGSIL	HGSIL	0	0.566930549	-1.183633057	2.317494155	0.816
Epitestosterone	NILM HPV−	NILM HPV+	0	-0.006862564	-1.572178166	1.558453037	1
	NILM HPV−	LGSIL	0	-0.584000593	-2.539079153	1.371077967	0.85
	NILM HPV−	HGSIL	0	0.628860086	-0.96466167	2.222381841	0.71
	NILM HPV+	LGSIL	0	-0.577138029	-2.402594023	1.248317966	0.827
	NILM HPV+	HGSIL	0	0.63572265	-0.795804157	2.067249457	0.629
	LGSIL	HGSIL	0	1.212860679	-0.636838821	3.062560179	0.303
Glyceric acid	NILM HPV−	NILM HPV+	0	-0.536940262	-1.587158104	0.51327758	0.518
	NILM HPV−	LGSIL	0	-0.387328828	-1.699050492	0.924392837	0.854
	NILM HPV−	HGSIL	0	0.404425978	-0.66471623	1.473568187	0.736
	NILM HPV+	LGSIL	0	0.149611434	-1.075142509	1.374365378	0.987
	NILM HPV+	HGSIL	0	0.94136624	-0.019088631	1.901821111	0.0564
	LGSIL	HGSIL	0	0.791754806	-0.449264842	2.032774455	0.326
Glycine	NILM HPV−	NILM HPV+	0	-0.307827948	-1.331879426	0.71622353	0.847
	NILM HPV−	LGSIL	0	-0.084027563	-1.363067449	1.195012323	0.998
	NILM HPV−	HGSIL	0	0.247134294	-0.795370046	1.289638634	0.917

	NILM HPV+	LGSIL	0	0.223800385	-0.970438596	1.418039366	0.957
	NILM HPV+	HGSIL	0	0.554962242	-0.381562725	1.491487209	0.39
	LGSIL	HGSIL	0	0.331161857	-0.878937566	1.54126128	0.88
Glycolic acid	NILM HPV−	NILM HPV+	0	-0.353308746	-1.364633104	0.658015612	0.78
	NILM HPV−	LGSIL	0	-0.165254038	-1.428397756	1.09788968	0.984
	NILM HPV−	HGSIL	0	0.297499819	-0.732048065	1.327047704	0.862
	NILM HPV+	LGSIL	0	0.188054708	-0.991342029	1.367451444	0.973
	NILM HPV+	HGSIL	0	0.650808565	-0.274077079	1.575694209	0.246
	LGSIL	HGSIL	0	0.462753857	-0.732306204	1.657813919	0.722
Histamine	NILM HPV−	NILM HPV+	0	-0.196017651	-1.501369958	1.109334655	0.977
	NILM HPV−	LGSIL	0	0.618973521	-1.011410987	2.249358029	0.734
	NILM HPV−	HGSIL	0	0.152619702	-1.176254358	1.481493761	0.989
	NILM HPV+	LGSIL	0	0.814991172	-0.707298128	2.337280473	0.478
	NILM HPV+	HGSIL	0	0.348637353	-0.845145432	1.542420138	0.858
	LGSIL	HGSIL	0	-0.466353819	-2.008860331	1.076152692	0.845
Hydroxypropionic acid	NILM HPV−	NILM HPV+	0	-0.391108573	-1.398231716	0.616014571	0.72
	NILM HPV−	LGSIL	0	-0.187262692	-1.445159096	1.070633711	0.977
	NILM HPV−	HGSIL	0	0.458980545	-0.566290421	1.484251512	0.623
	NILM HPV+	LGSIL	0	0.20384588	-0.97065144	1.378343201	0.965
	NILM HPV+	HGSIL	0	0.850089118	-0.070954393	1.771132629	0.079
	LGSIL	HGSIL	0	0.646243238	-0.54385234	1.836338815	0.466
Hypoxanthine	NILM HPV−	NILM HPV+	0	-0.391694397	-1.390835352	0.607446558	0.715
	NILM HPV−	LGSIL	0	-0.358330268	-1.606256921	0.889596385	0.864
	NILM HPV−	HGSIL	0	0.369750822	-0.647394121	1.386895766	0.759

	NILM HPV+	LGSIL	0	0.033364129	-1.13182444	1.198552698	1
	NILM HPV+	HGSIL	0	0.76144522	-0.152298347	1.675188786	0.13
	LGSIL	HGSIL	0	0.728081091	-0.452582108	1.908744289	0.355
Isoleucine	NILM HPV−	NILM HPV+	0	-0.241755883	-1.277455795	0.79394403	0.921
	NILM HPV−	LGSIL	0	0.063082965	-1.230505811	1.356671742	0.999
	NILM HPV−	HGSIL	0	0.537550248	-0.516812425	1.591912922	0.52
	NILM HPV+	LGSIL	0	0.304838848	-0.902984426	1.512662122	0.902
	NILM HPV+	HGSIL	0	0.779306131	-0.16787167	1.726483931	0.137
	LGSIL	HGSIL	0	0.474467283	-0.749396843	1.698331409	0.721
L-3-Phenyllactic acid	NILM HPV−	NILM HPV+	0	-0.244605705	-1.443244139	0.954032729	0.945
	NILM HPV−	LGSIL	0	-0.224788463	-1.721887387	1.272310461	0.977
	NILM HPV−	HGSIL	0	0.553157309	-0.667079951	1.773394569	0.614
	NILM HPV+	LGSIL	0	0.019817242	-1.378023368	1.417657851	1
	NILM HPV+	HGSIL	0	0.797763014	-0.29842682	1.893952848	0.22
	LGSIL	HGSIL	0	0.777945772	-0.638459271	2.194350815	0.456
L-Alanine	NILM HPV−	NILM HPV+	0	-0.420312345	-1.362726426	0.522101736	0.626
	NILM HPV−	LGSIL	0	-0.137874503	-1.314949313	1.039200307	0.989
	NILM HPV−	HGSIL	0	0.445940818	-0.513455063	1.405336699	0.595
	NILM HPV+	LGSIL	0	0.282437843	-0.816596392	1.381472077	0.898
	NILM HPV+	HGSIL	0	0.866253163	0.004387979	1.728118348	0.0485
	LGSIL	HGSIL	0	0.58381532	-0.529814961	1.697445602	0.496
L-Asparagine	NILM HPV−	NILM HPV+	0	0.089208228	-1.369608933	1.548025388	0.998
	NILM HPV−	LGSIL	0	-0.670837064	-2.492899114	1.151224985	0.752
	NILM HPV−	HGSIL	0	0.526253278	-0.958850991	2.011357547	0.773

	NILM HPV+	LGSIL	0	-0.760045292	-2.46130383	0.941213245	0.625
	NILM HPV+	HGSIL	0	0.437045051	-0.897085823	1.771175924	0.811
	LGSIL	HGSIL	0	1.197090343	-0.526762259	2.920942945	0.256
L-Aspartic acid	NILM HPV−	NILM HPV+	0	-0.331646284	-1.392664421	0.729371854	0.832
	NILM HPV−	LGSIL	0	-0.039052815	-1.364264043	1.286158414	1
	NILM HPV−	HGSIL	0	0.389181385	-0.690955735	1.469318504	0.764
	NILM HPV+	LGSIL	0	0.292593469	-0.944755675	1.529942612	0.918
	NILM HPV+	HGSIL	0	0.720827668	-0.249504388	1.691159724	0.205
	LGSIL	HGSIL	0	0.428234199	-0.825547924	1.682016322	0.792
L-Cysteine	NILM HPV−	NILM HPV+	0	-0.512669438	-1.851475453	0.826136577	0.729
	NILM HPV−	LGSIL	0	-0.53208574	-2.204253917	1.140082436	0.824
	NILM HPV−	HGSIL	0	-0.039873488	-1.402804074	1.323057099	1
	NILM HPV+	LGSIL	0	-0.019416302	-1.580718996	1.541886392	1
	NILM HPV+	HGSIL	0	0.47279595	-0.751581228	1.697173129	0.724
	LGSIL	HGSIL	0	0.492212252	-1.089825781	2.074250286	0.834
L-Glutamic acid	NILM HPV−	NILM HPV+	0	-0.374670048	-1.424235091	0.674894995	0.769
	NILM HPV−	LGSIL	0	-0.420833111	-1.73173943	0.890073207	0.82
	NILM HPV−	HGSIL	0	0.134702486	-0.93377516	1.203180132	0.986
	NILM HPV+	LGSIL	0	-0.046163063	-1.270155719	1.177829592	1
	NILM HPV+	HGSIL	0	0.509372534	-0.450485333	1.469230401	0.486
	LGSIL	HGSIL	0	0.555535597	-0.684712653	1.795783847	0.623
L-Glutamine	NILM HPV−	NILM HPV+	0	-0.247553379	-1.7243851	1.229278342	0.968
	NILM HPV−	LGSIL	0	-0.281314261	-2.125876489	1.563247967	0.976
	NILM HPV−	HGSIL	0	-0.613031324	-2.116474766	0.890412118	0.689

	NILM HPV+	LGSIL	0	-0.033760882	-1.756027826	1.688506062	1
	NILM HPV+	HGSIL	0	-0.365477945	-1.71608366	0.98512777	0.883
	LGSIL	HGSIL	0	-0.331717063	-2.07685708	1.413422954	0.955
L-Leucine	NILM HPV−	NILM HPV+	0	-0.289800938	-1.315430801	0.735828926	0.869
	NILM HPV−	LGSIL	0	-0.053814815	-1.334826104	1.227196474	0.999
	NILM HPV−	HGSIL	0	0.349367929	-0.694743239	1.393479096	0.801
	NILM HPV+	LGSIL	0	0.235986123	-0.960093557	1.432065802	0.95
	NILM HPV+	HGSIL	0	0.639168867	-0.29879958	1.577137313	0.271
	LGSIL	HGSIL	0	0.403182744	-0.808781823	1.615147311	0.804
L-Lysine	NILM HPV−	NILM HPV+	0	-0.580751663	-2.104796462	0.943293136	0.732
	NILM HPV−	LGSIL	0	-0.659951329	-2.563482673	1.243580014	0.784
	NILM HPV−	HGSIL	0	0.198669662	-1.352837613	1.750176937	0.985
	NILM HPV+	LGSIL	0	-0.079199667	-1.856526048	1.698126715	0.999
	NILM HPV+	HGSIL	0	0.779421325	-0.614362128	2.173204778	0.441
	LGSIL	HGSIL	0	0.858620991	-0.942309696	2.659551679	0.575
L-Methionine	NILM HPV−	NILM HPV+	0	-0.368275315	-1.432601014	0.696050384	0.785
	NILM HPV−	LGSIL	0	-0.219800157	-1.549142529	1.109542214	0.97
	NILM HPV−	HGSIL	0	0.453599785	-0.629904497	1.537104066	0.671
	NILM HPV+	LGSIL	0	0.148475158	-1.092731232	1.389681548	0.988
	NILM HPV+	HGSIL	0	0.821875099	-0.151481818	1.795232017	0.122
	LGSIL	HGSIL	0	0.673399942	-0.584290655	1.931090538	0.478
L-Ornithine	NILM HPV−	NILM HPV+	0	-0.874955839	-2.417949475	0.668037796	0.428
	NILM HPV−	LGSIL	0	-0.169152225	-2.096350658	1.758046208	0.995
	NILM HPV−	HGSIL	0	-0.613210475	-2.184008034	0.957587084	0.717

	NILM HPV+	LGSIL	0	0.705803615	-1.093620718	2.505227947	0.714
	NILM HPV+	HGSIL	0	0.261745364	-1.149367353	1.672858081	0.958
	LGSIL	HGSIL	0	-0.44405825	-2.267380367	1.379263866	0.911
L-Phenylalanine	NILM HPV−	NILM HPV+	0	-0.32010843	-1.360360174	0.720143314	0.838
	NILM HPV−	LGSIL	0	-0.064167512	-1.363441523	1.2351065	0.999
	NILM HPV−	HGSIL	0	0.263432933	-0.795563594	1.322429459	0.906
	NILM HPV+	LGSIL	0	0.255940918	-0.957190658	1.469072493	0.94
	NILM HPV+	HGSIL	0	0.583541363	-0.36779922	1.534881946	0.36
	LGSIL	HGSIL	0	0.327600445	-0.901642482	1.556843371	0.888
L-Proline	NILM HPV−	NILM HPV+	0	-0.357697841	-1.580813839	0.865418157	0.857
	NILM HPV−	LGSIL	0	-0.107879033	-1.635550425	1.419792359	0.997
	NILM HPV−	HGSIL	0	0.020698993	-1.224456904	1.26585489	1
	NILM HPV+	LGSIL	0	0.249818808	-1.176567301	1.676204918	0.964
	NILM HPV+	HGSIL	0	0.378396834	-0.740178447	1.496972115	0.796
	LGSIL	HGSIL	0	0.128578025	-1.316751624	1.573907675	0.995
L-Serine	NILM HPV−	NILM HPV+	0	-0.540422779	-1.569971998	0.48912644	0.495
	NILM HPV−	LGSIL	0	-0.194713599	-1.480620161	1.091192963	0.976
	NILM HPV−	HGSIL	0	0.070964794	-0.977136354	1.119065941	0.998
	NILM HPV+	LGSIL	0	0.34570918	-0.854941213	1.546359574	0.863
	NILM HPV+	HGSIL	0	0.611387573	-0.330165239	1.552940384	0.311
	LGSIL	HGSIL	0	0.265678392	-0.950917592	1.482274377	0.934
L-Threonine	NILM HPV−	NILM HPV+	0	-0.339194644	-1.375168064	0.696778776	0.812
	NILM HPV−	LGSIL	0	0.031792938	-1.26213745	1.325723325	1
	NILM HPV−	HGSIL	0	0.522328942	-0.532312167	1.576970052	0.544

	NILM HPV+	LGSIL	0	0.370987582	-0.837154654	1.579129817	0.839
	NILM HPV+	HGSIL	0	0.861523586	-0.085904345	1.808951517	0.0854
	LGSIL	HGSIL	0	0.490536005	-0.733651319	1.714723329	0.701
L-Tryptophan	NILM HPV−	NILM HPV+	0	-0.861099546	-2.386846671	0.664647579	0.433
	NILM HPV−	LGSIL	0	0.092098674	-1.813558875	1.997756222	0.999
	NILM HPV−	HGSIL	0	-0.053574274	-1.60681455	1.499666002	1
	NILM HPV+	LGSIL	0	0.95319822	-0.826113398	2.732509838	0.478
	NILM HPV+	HGSIL	0	0.807525272	-0.587815008	2.202865552	0.411
	LGSIL	HGSIL	0	-0.145672947	-1.948615237	1.657269342	0.996
L-Tyrosine	NILM HPV−	NILM HPV+	0	-0.492861855	-1.506828279	0.521104568	0.559
	NILM HPV−	LGSIL	0	-0.141291742	-1.407735398	1.125151915	0.99
	NILM HPV−	HGSIL	0	0.12125738	-0.910980179	1.153494938	0.989
	NILM HPV+	LGSIL	0	0.351570114	-0.830907774	1.534048001	0.851
	NILM HPV+	HGSIL	0	0.614119235	-0.313182655	1.541421125	0.295
	LGSIL	HGSIL	0	0.262549121	-0.935633012	1.460731254	0.933
L-Valine	NILM HPV−	NILM HPV+	0	-0.609746934	-1.740895552	0.521401685	0.472
	NILM HPV−	LGSIL	0	-0.218038071	-1.630842243	1.194766101	0.975
	NILM HPV−	HGSIL	0	0.311110242	-0.840421073	1.462641557	0.884
	NILM HPV+	LGSIL	0	0.391708863	-0.927425774	1.710843499	0.852
	NILM HPV+	HGSIL	0	0.920857176	-0.113611253	1.955325604	0.0951
	LGSIL	HGSIL	0	0.529148313	-0.807505479	1.865802105	0.708
L-alpha-Aminobutyric acid	NILM HPV−	NILM HPV+	0	-0.484195687	-2.017124649	1.048733274	0.827
	NILM HPV−	LGSIL	0	-0.179637144	-2.094264803	1.734990516	0.994
	NILM HPV−	HGSIL	0	0.187047752	-1.373503773	1.747599277	0.988

	NILM HPV+	LGSIL	0	0.304558543	-1.483128463	2.09224555	0.967
	NILM HPV+	HGSIL	0	0.671243439	-0.73066484	2.073151718	0.571
	LGSIL	HGSIL	0	0.366684896	-1.444744014	2.178113805	0.946
Lactic acid	NILM HPV−	NILM HPV+	0	-0.545170001	-1.596072522	0.505732519	0.505
	NILM HPV−	LGSIL	0	-0.277545073	-1.5901219	1.035031755	0.939
	NILM HPV−	HGSIL	0	-0.08824799	-1.158087214	0.981591235	0.996
	NILM HPV+	LGSIL	0	0.267624929	-0.95792748	1.493177337	0.934
	NILM HPV+	HGSIL	0	0.456922012	-0.504159017	1.418003041	0.577
	LGSIL	HGSIL	0	0.189297083	-1.052531635	1.431125801	0.976
Malic acid	NILM HPV−	NILM HPV+	0	-0.496926894	-1.460769083	0.466915295	0.51
	NILM HPV−	LGSIL	0	-0.359931793	-1.563770301	0.843906716	0.849
	NILM HPV−	HGSIL	0	0.582085058	-0.399125055	1.56329517	0.389
	NILM HPV+	LGSIL	0	0.136995101	-0.987028387	1.261018589	0.987
	NILM HPV+	HGSIL	0	1.079011951	0.197550136	1.960473766	0.0116
	LGSIL	HGSIL	0	0.94201685	-0.196934562	2.080968263	0.134
N-Acetyl-L-glutamic acid	NILM HPV−	NILM HPV+	0	-0.292164085	-1.481709403	0.897381233	0.909
	NILM HPV−	LGSIL	0	-0.664919735	-2.150661362	0.820821891	0.624
	NILM HPV−	HGSIL	0	0.527398522	-0.68358177	1.738378813	0.644
	NILM HPV+	LGSIL	0	-0.372755651	-1.759991956	1.014480655	0.885
	NILM HPV+	HGSIL	0	0.819562607	-0.268311308	1.907436521	0.194
	LGSIL	HGSIL	0	1.192318257	-0.213341648	2.597978163	0.12
N-Acetylaspartic acid	NILM HPV−	NILM HPV+	0	-0.350078425	-1.385517699	0.685360849	0.796
	NILM HPV−	LGSIL	0	-0.025210074	-1.318473313	1.268053165	1
	NILM HPV−	HGSIL	0	0.514724912	-0.539372426	1.568822251	0.555

	NILM HPV+	LGSIL	0	0.324868351	-0.882650968	1.53238767	0.885
	NILM HPV+	HGSIL	0	0.864803338	-0.082136101	1.811742777	0.0835
	LGSIL	HGSIL	0	0.539934987	-0.683621148	1.763491122	0.634
N-Acetylneuraminic acid	NILM HPV−	NILM HPV+	0	0.084891855	-1.282050372	1.451834082	0.998
	NILM HPV−	LGSIL	0	-0.429360015	-2.136670309	1.277950279	0.903
	NILM HPV−	HGSIL	0	-0.910128958	-2.301702756	0.481444841	0.305
	NILM HPV+	LGSIL	0	-0.51425187	-2.108366744	1.079863004	0.818
	NILM HPV+	HGSIL	0	-0.995020813	-2.245129378	0.255087753	0.157
	LGSIL	HGSIL	0	-0.480768943	-2.096054928	1.134517043	0.851
Niacinamide	NILM HPV−	NILM HPV+	0	0.037894315	-1.376919051	1.45270768	1
	NILM HPV−	LGSIL	0	0.198618784	-1.568482543	1.965720111	0.99
	NILM HPV−	HGSIL	0	-0.255050115	-1.695357664	1.185257435	0.963
	NILM HPV+	LGSIL	0	0.160724469	-1.489217266	1.810666204	0.993
	NILM HPV+	HGSIL	0	-0.292944429	-1.586832548	1.00094369	0.927
	LGSIL	HGSIL	0	-0.453668899	-2.12552317	1.218185373	0.882
Phosphoric acid	NILM HPV−	NILM HPV+	0	-0.474594068	-1.500277594	0.551089457	0.598
	NILM HPV−	LGSIL	0	-0.199880566	-1.480958879	1.081197746	0.974
	NILM HPV−	HGSIL	0	0.160354008	-0.883811788	1.204519805	0.975
	NILM HPV+	LGSIL	0	0.274713502	-0.921428758	1.470855761	0.924
	NILM HPV+	HGSIL	0	0.634948076	-0.303069446	1.572965598	0.277
	LGSIL	HGSIL	0	0.360234575	-0.851793404	1.572262553	0.851
Putrescine	NILM HPV−	NILM HPV+	0	-0.512163657	-2.113844888	1.089517574	0.822
	NILM HPV−	LGSIL	0	-0.308848521	-2.309347738	1.691650696	0.975
	NILM HPV−	HGSIL	0	0.525110281	-1.105432393	2.155652955	0.819

	NILM HPV+	LGSIL	0	0.203315136	-1.664550106	2.071180378	0.991
	NILM HPV+	HGSIL	0	1.037273938	-0.427510298	2.502058175	0.241
	LGSIL	HGSIL	0	0.833958802	-1.058713174	2.726630778	0.635
Tetradecanedioic acid	NILM HPV−	NILM HPV+	0	-0.590182845	-1.877880071	0.697514381	0.606
	NILM HPV−	LGSIL	0	0.080934905	-1.527398415	1.689268225	0.999
	NILM HPV−	HGSIL	0	0.212237619	-1.098663226	1.523138463	0.971
	NILM HPV+	LGSIL	0	0.67111775	-0.830582366	2.172817867	0.625
	NILM HPV+	HGSIL	0	0.802420464	-0.375216235	1.980057163	0.271
	LGSIL	HGSIL	0	0.131302714	-1.390341173	1.6529466	0.995
Tyramine	NILM HPV−	NILM HPV+	0	-0.14015148	-1.904928824	1.624625865	0.996
	NILM HPV−	LGSIL	0	-0.184697664	-2.38890386	2.019508533	0.996
	NILM HPV−	HGSIL	0	0.578245778	-1.218331914	2.374823471	0.819
	NILM HPV+	LGSIL	0	-0.044546184	-2.102612544	2.013520176	1
	NILM HPV+	HGSIL	0	0.718397258	-0.895543134	2.33233765	0.628
	LGSIL	HGSIL	0	0.762943442	-1.322455674	2.848342558	0.755
Uracil	NILM HPV−	NILM HPV+	0	-0.135058724	-1.306762534	1.036645087	0.989
	NILM HPV−	LGSIL	0	-0.61069261	-2.0741502	0.85276498	0.674
	NILM HPV−	HGSIL	0	0.505413462	-0.687403827	1.698230751	0.663
	NILM HPV+	LGSIL	0	-0.475633886	-1.842063597	0.890795824	0.782
	NILM HPV+	HGSIL	0	0.640472186	-0.431085149	1.71202952	0.382
	LGSIL	HGSIL	0	1.116106072	-0.26847091	2.500683054	0.149
Urea	NILM HPV−	NILM HPV+	0	-0.567290076	-2.046168008	0.911587856	0.728
	NILM HPV−	LGSIL	0	-0.245492499	-2.092610445	1.601625446	0.984
	NILM HPV−	HGSIL	0	-0.426811631	-1.932338157	1.078714894	0.868

	NILM HPV+	LGSIL	0	0.321797577	-1.402855639	2.046450793	0.957
	NILM HPV+	HGSIL	0	0.140478445	-1.21199859	1.49295548	0.992
	LGSIL	HGSIL	0	-0.181319132	-1.928877113	1.566238848	0.992
Uric acid	NILM HPV−	NILM HPV+	0	-0.41745191	-1.495992175	0.661088356	0.722
	NILM HPV−	LGSIL	0	-0.256692659	-1.603789019	1.0904037	0.955
	NILM HPV−	HGSIL	0	-0.052063126	-1.150038112	1.045911861	0.999
	NILM HPV+	LGSIL	0	0.16075925	-1.09702403	1.418542531	0.985
	NILM HPV+	HGSIL	0	0.365388784	-0.62096777	1.351745337	0.748
	LGSIL	HGSIL	0	0.204629533	-1.069858108	1.479117175	0.972

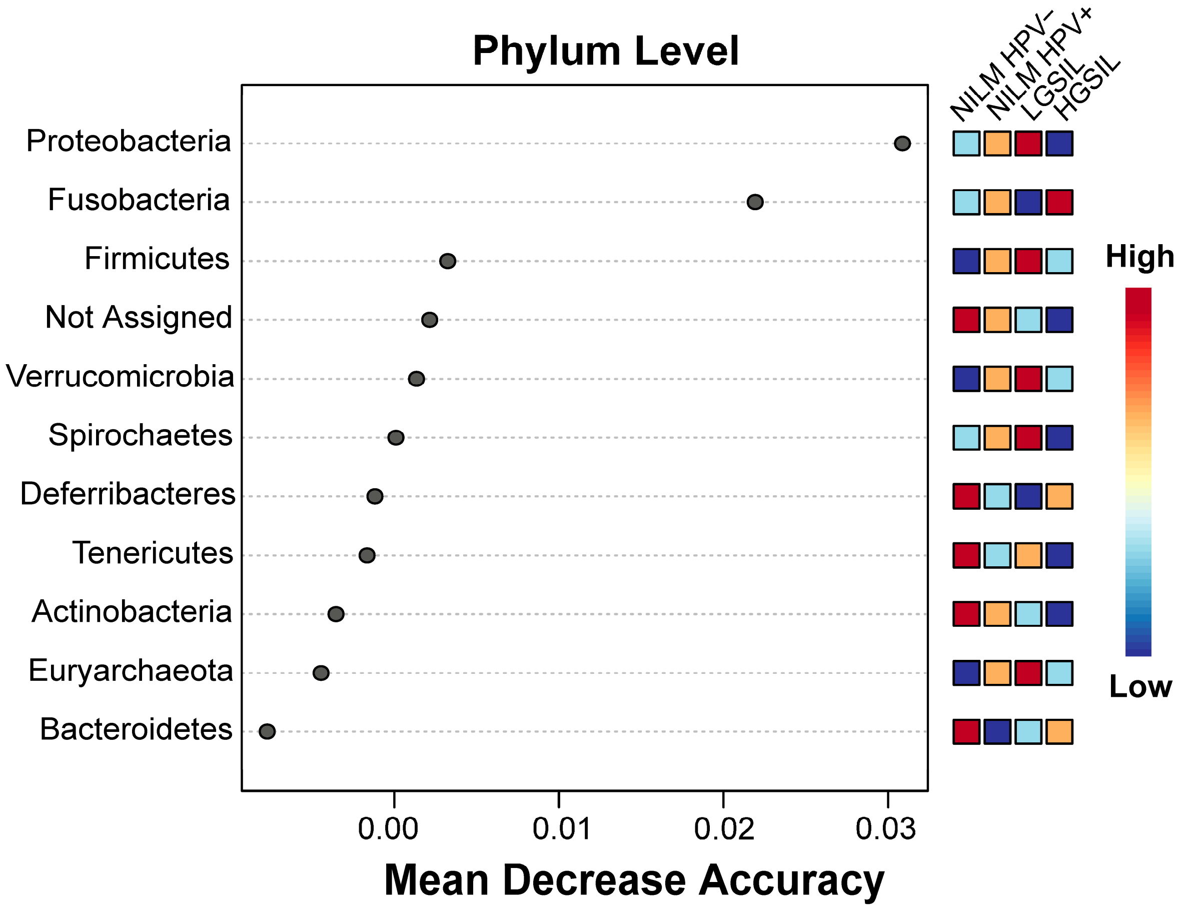

Figure S1: Exploratory phylum-level Random Forest analysis of patients with cervical disease. Fusobacteria was identified as a potential contributor to the HGSIL group classification, while Proteobacteria was observed for the LGSIL group.

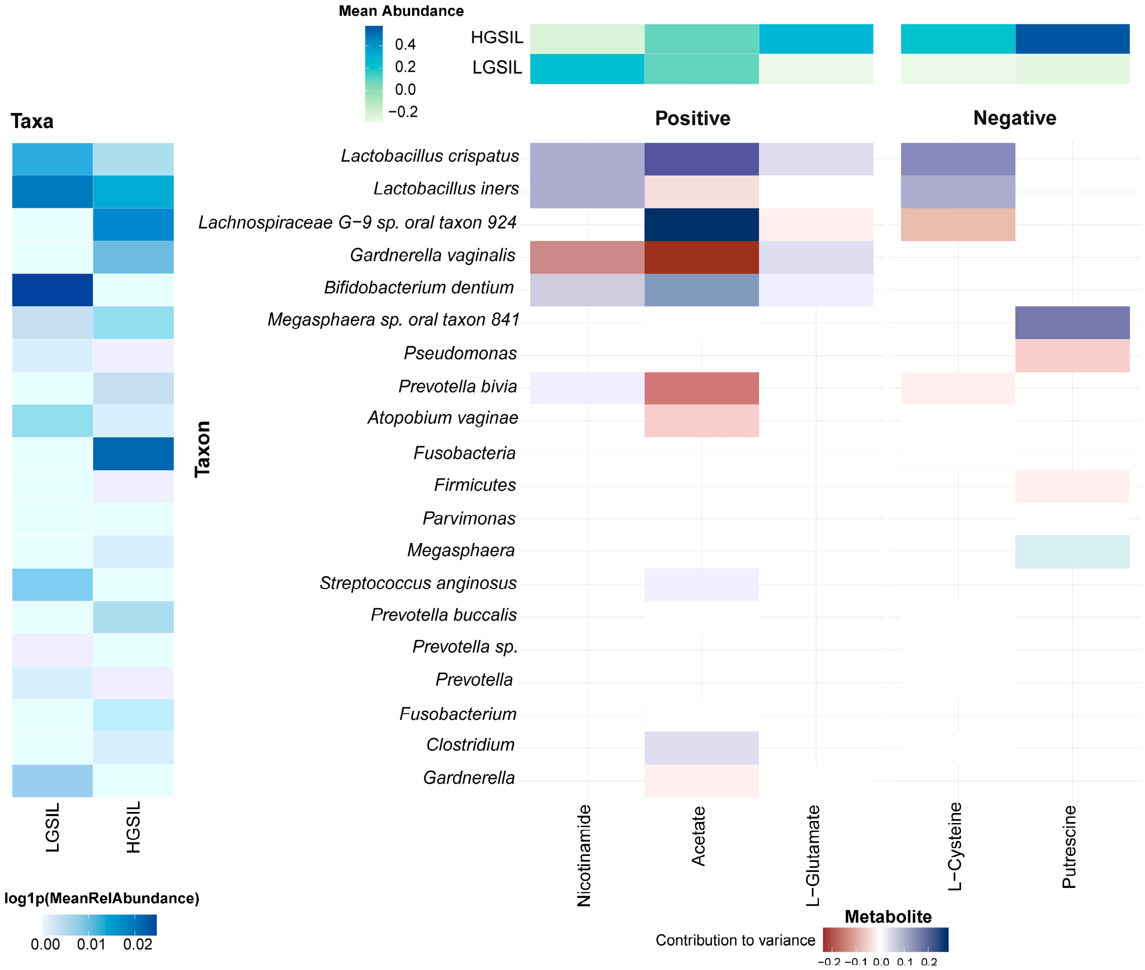

Figure S2: Diverse taxa explain biogenic amine variation across cervical dysplasia progression. Concentration abundance of the biogenic amine putrescine across cervical phenotypes shows that patients with HGSIL had the highest abundance when compared with patients with low-grade lesions. Putrescine was identified as an important differential metabolite across groups (p-value<0.1; VarShare ± 0.01). Differences in the relative capacity of community members to synthesize putrescine identified by MIMOSA2 showed *Pseudomonas* and *Parvimonas* as the highest contributors, whereas degradation of putrescine was driven by *Pseudomonas*, *Parvimonas*, and *Megasphaera oral taxon 841*. Analyzed groups included LGSIL (n = 5) and HGSIL (n = 11).

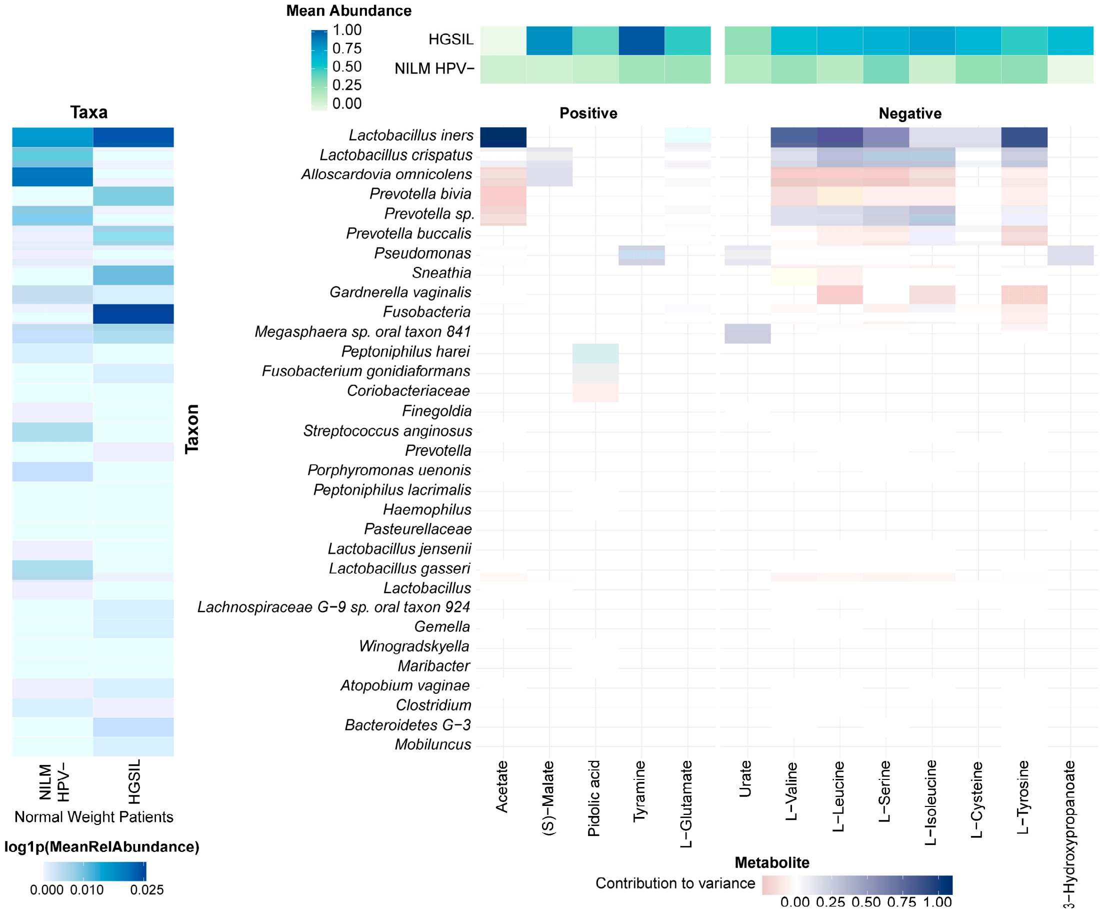

Figure S3: Tyramine degradation remains a significant pathway in cervical dysplasia regardless of BMI. Analyzed groups included normal weight patients classified as NILM HPV− (n = 6) and HGSIL (n = 4).

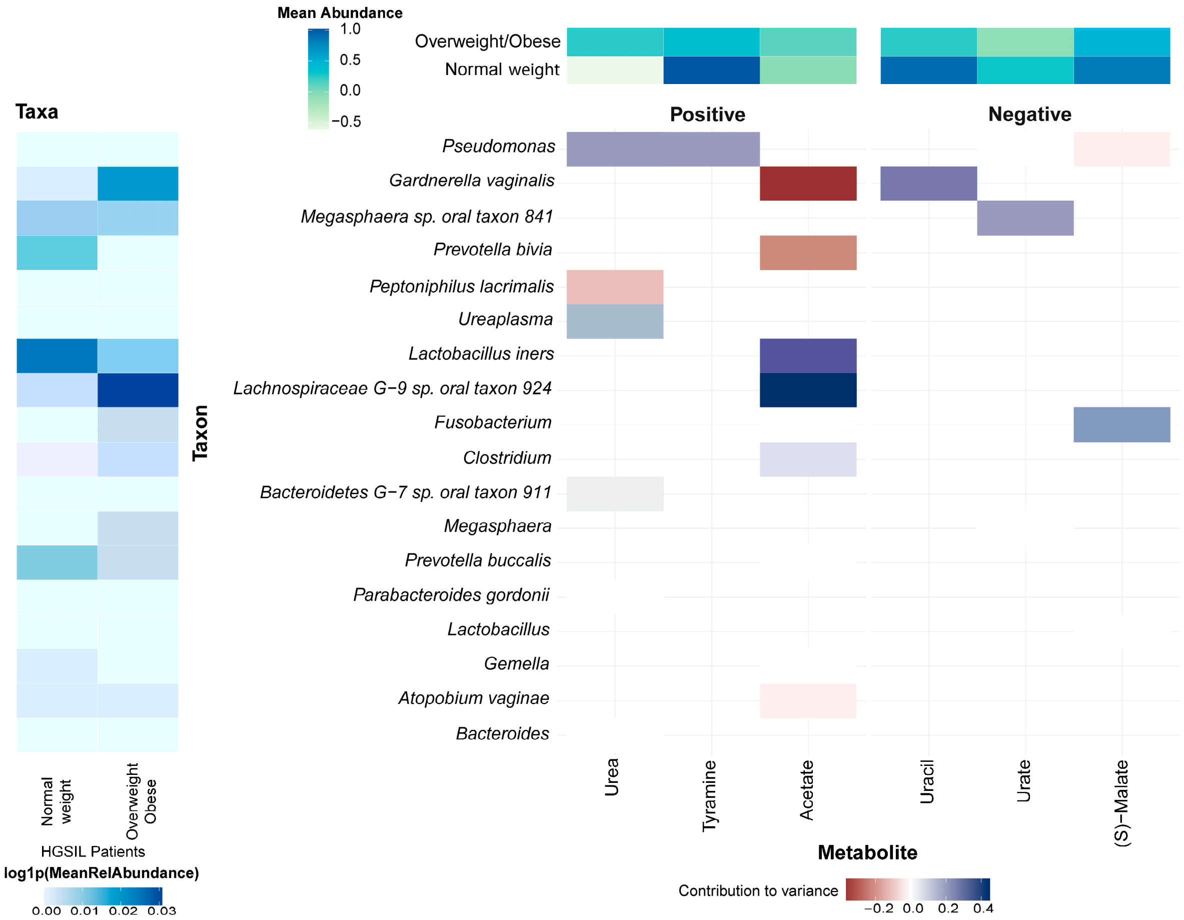

Figure S4: Tyramine degradation is a significant pathway when comparing normal and overweight/obese HGSIL patients. *Pseudomonas* was associated with tyramine and urea metabolism when comparing patients with high-severity lesions across BMI classifications. Taxa commonly found in the oral cavity remained as potential contributors of metabolic differences across the groups. Analyzed groups included HGSIL patients classified as normal weight (n = 4) and obese/overweight (n = 6).
